# SMBO: a Sinusoidal Modulation Bi-population Optimizer for brain cancer MRI image clustering

**DOI:** 10.3389/fnins.2026.1849626

**Published:** 2026-09-30

**Authors:** Yingzhi Li, Kan Yu, Zhiwei Ye

**Affiliations:** 1 Faculty of Information Science and Technology, Wenhua College, Wuhan, Hubei, China; 2 School of Computer Science and Artificial Intelligence, Hubei University of Technology, Wuhan, Hubei, China

**Keywords:** bi-population strategy, brain cancer clustering, MRI image analysis, optimization, sinusoidal modulation

## Abstract

This study proposes the Sinusoidal Modulation Bi-population Optimizer (SMBO), which couples a sinusoidal modulation of the step size, jointly driven by iteration progress and individual fitness quality, with dynamic behavior switching (DBS) to achieve a self-adaptive, threshold-free balance between exploration and exploitation. This balance is further reinforced by an asymmetric information flow (AIF) mechanism that sustains diversity between the elite and exploration sub-populations. In simulation experiments on the CEC2017 benchmark suite, SMBO achieves the lowest average rank among six compared algorithms at both *D* = 10 (1.38) and *D* = 100 (1.10), and on CEC2022 at *D* = 10 (1.33). Friedman and Wilcoxon rank-sum tests confirm that these differences are statistically significant. Applied to 4-class brain tumor MRI clustering across three deep feature extraction backbones (ResNet50, VGG16, DenseNet121) combined with PCA, SMBO consistently achieves the best clustering purity, normalized mutual information, and adjusted Rand index among all compared algorithms, reaching a purity of 0.8636, an NMI of 0.6733, and an ARI of 0.6881 with the DenseNet121 backbone. These results establish SMBO as a competitive optimizer for both numerical benchmarks and medical image analysis pipelines.

## 1 Introduction

Cancer remains one of the most devastating diseases worldwide, imposing a severe burden on both patients and healthcare systems. Among all malignancies, brain cancer is particularly lethal: approximately 70% of patients diagnosed with this disease do not survive (Mercaldo et al., 2023a), and delayed or inaccurate diagnosis directly reduces the likelihood of effective treatment. Early and precise grouping of tumor type is therefore a clinical imperative, as different tumor subtypes—including glioma, meningioma, and pituitary tumors—require fundamentally different therapeutic strategies (Srinivasan et al., 2024). Magnetic resonance imaging (MRI) has become the primary modality for brain tumor examination due to its superior soft-tissue contrast, yet manual interpretation of MRI scans is labor-intensive, subject to inter-observer variability, and heavily dependent on the experience and fatigue state of the radiologist (Hossain et al., 2024). These limitations underscore the urgent need for automated, reliable, and accurate computer-aided diagnosis (CAD) systems that support clinical decision-making at scale.

Deep learning has emerged as the dominant paradigm for medical image classification, with convolutional neural networks (CNNs) and, more recently, vision transformers achieving state-of-the-art performance across a range of cancer imaging tasks. In the context of brain tumor classification, a variety of architectures have been explored, ranging from lightweight residual multiscale networks (Dutta et al., 2024) and attention-based hybrid CNN-transformer models (Goceri, 2024) to transfer-learning ensembles (Kang et al., 2021) and randomized vision transformers (Wang et al., 2024). Similar advances have been reported for other cancer types: breast cancer clustering has been approached through ensemble deep CNNs with segmentation modules (Islam et al., 2024b), multiscale texture analysis (Guo et al., 2024b), and CNN-LSTM hybrid architectures (Kaddes et al., 2025); skin lesion classification has been addressed through multi-scale attention mechanisms combined with ensemble learning (Yang et al., 2025); and lung cancer subtype identification has benefited from transformer-based optimization (Yagappan et al., 2025). Despite these impressive results, deep learning models are highly sensitive to the choice of hyperparameters, including learning rate, batch size, network depth, and regularization coefficients. Suboptimal hyperparameter configurations routinely lead to overfitting, poor generalization, and degraded clustering accuracy—a challenge that is particularly acute when labeled medical imaging data are scarce.

Metaheuristic optimization algorithms offer a gradient-free, population-based solution to the hyperparameter selection problem, requiring no assumption on the smoothness or convexity of the objective landscape (Wan et al., 2016; Ye et al., 2023; Wang et al., 2017). A huge amount of nature-inspired metaheuristic algorithms have been proposed in the literature (Ma et al., 2023; Mansouri et al., 2025), reflecting the breadth of research interest in this area. Representative recent proposals include the Horned Lizard Optimization Algorithm (Peraza-Vázquez et al., 2024), the Snow Ablation Optimizer (Deng and Liu, 2023), the Political Optimizer (Askari et al., 2020), and the Dream Optimization Algorithm (Lang and Gao, 2025), all of which demonstrate competitive performance on standard numerical benchmarks.

Although differential evolution (DE) and its variants, such as Success-History based Adaptive Differential Evolution (SHADE) and its linear population size reduction extension (L-SHADE), have demonstrated excellent optimization capability (Tanabe and Fukunaga, 2013; Piotrowski, 2018; Tanabe and Fukunaga, 2014), optimization problems grounded in real-world modeling remain a persistent challenge in this field.

A persistent challenge across these methods is the balance between exploration of the global search space and exploitation of promising local regions: premature convergence to local optima is a common failure mode, especially in high-dimensional settings (Guo et al., 2023c). A series of prior work has progressively addressed this issue through dynamic reconstruction (Guo and Sato, 2018), fission-fusion hybridization (Guo and Sato, 2019), twinning bare-bones strategies (Guo et al., 2022), twinning memory mechanisms (Xiao et al., 2022), crossed-memory offspring selection (Guo et al., 2023a), pair barracuda swarm (Guo et al., 2023b), deep backtracking bare-bones search (Guo et al., 2025), and snow leopard territorial behavior (Guo et al., 2024a).

Multi-population strategies (Ma et al., 2019) have become a widely adopted design paradigm in nature-inspired optimization because they address several structural weaknesses of single-population metaheuristics. By partitioning the search effort across two or more interacting sub-populations, these methods can decouple exploration from exploitation. This partitioning also mitigates premature convergence: sub-populations evolve semi-independently, so the loss of diversity in one sub-population does not necessarily propagate to the others. Information-exchange mechanisms (migration, elite sharing, and archive-based transfer) allow good solutions to be reintroduced without collapsing the entire population onto a single local optimum.

Motivated by the clinical urgency of brain cancer diagnosis and the limitations of existing optimization strategies, this study proposes the Sinusoidal Modulation Bi-population Optimizer (SMBO), which couples a sinusoidal modulation step size with dynamic behavior switching (DBS)and asymmetric information flow (AIF) to achieve a principled, self-adaptive balance between exploration and exploitation. We evaluate the algorithm on the CEC benchmark suite to assess its numerical optimization performance, then apply it to clustering brain cancer MRI images to demonstrate its practical utility in a high-stakes clinical task. The main contributions of this work are as follows:

- A sinusoidal modulation step size Φ(*p, f*_*i*_) that jointly couples iteration progress and individual fitness quality, providing a unified and self-adaptive step size for exploitation stages.
- An asymmetric information flow mechanism that enables directed knowledge transfer between the elite convergence pool and the diversity exploration pool without symmetrical interference.
- A dynamic behavior switching scheme that probabilistically drives the algorithm from exploration to exploitation as a smooth function of iteration progress, eliminating the need for manual stage thresholds.
- Competitive performance on CEC2017 and CEC2022 benchmark functions and superior clustering quality on brain cancer MRI clustering compared to representative state-of-the-art methods.

The remainder of this study is organized as follows. Section 2 reviews related work on brain cancer image clustering and metaheuristic optimization. Section 3 presents the proposed SMBO algorithm in detail. Section 4 reports experimental results on both benchmark functions and the brain cancer clustering task. Section 5 discusses these results in relation to SMBO's core mechanisms and outlines the study's limitations. Section 6 concludes the paper.

## 2 Related work

### 2.1 Brain cancer detection and classification

Early and accurate detection of brain tumors from MRI scans is a prerequisite for effective treatment. Microwave imaging sensors have been explored as a complementary modality: (Hamza et al. 2024) designed a metamaterial-based microstrip sensor that achieves 8.5 dBi realized gain and demonstrates tumor-size discrimination in phantom experiments. For image-based classification, convolutional neural networks have dominated the literature. (Mercaldo et al. 2023b) combined VGG16, ResNet50, AlexNet, and MobileNet with class activation mapping, achieving detection accuracies ranging from 97.83% to 99.67% and providing spatial localization of cancerous regions. (Akter et al. 2024) proposed a deep CNN architecture paired with a U-Net segmentation model, reporting a peak classification accuracy of 98.8% on merged MRI datasets. (Nagarani et al. 2024) incorporated a self-attention-based progressive generative adversarial network optimized with a momentum search algorithm, achieving accuracy gains of over 11% relative to comparable baselines.

Hybrid architectures that combine deep feature extraction with classical machine learning classifiers have also attracted considerable attention. (Celik and Inik 2024) proposed a CNN-based feature extractor coupled with Bayesian-optimized support vector machines, achieving 97.15% mean classification accuracy while also demonstrating competitive time efficiency. (Shoaib et al. 2025) evaluated DenseNet201, EfficientNetB5, and InceptionResNetV2 as feature extractors followed by principal component analysis and multiple machine learning classifiers, with DenseNet201 combined with SVM reaching 100% accuracy on one dataset. (Allahem et al. 2025) similarly combined vision transformer feature extraction with PCA dimensionality reduction and random forest classification, attaining 99% accuracy on the Brain Tumor MRI dataset. (Hossain et al. 2024) constructed an ensemble of transfer learning models—including VGG16, InceptionV3, and Xception—augmented with explainable AI and vision transformers, reaching a peak ensemble accuracy of 96.94%.

More recent research has explored fine-grained architectural refinements. (Reddy et al. 2024) evaluated fine-tuned vision transformer variants (FTVT), with FTVT-l16 achieving 98.70% accuracy on a four-class MRI dataset comprising glioma, meningioma, pituitary, and no-tumor cases. (Islam et al. 2024a) proposed BrainNet, an optimized EfficientNet architecture that reached 99.69% accuracy on T1-weighted CE-MRI images. (Dutta et al. 2024) designed ARM-Net, an attention-guided residual multiscale CNN with a lightweight global attention module, achieving 97.11% accuracy on the BraTS 2020 dataset. (Srinivasan et al. 2024) presented three distinct CNN models for tumor detection, five-class categorization, and grade classification, respectively, employing grid search for hyperparameter tuning and reaching 99.53% detection accuracy.

### 2.2 Medical image clustering beyond brain tumors

Automated clustering of medical images extends well beyond brain cancer. In breast cancer diagnosis, (Ting et al. 2019) proposed an improved CNN architecture for mammographic clustering into malignant, benign, and healthy categories, establishing an early benchmark for deep learning in this domain. (Murtaza et al. 2020) provided a systematic review of deep learning methods for breast cancer clustering across multiple imaging modalities, identifying ten open research challenges for the field. (Islam et al. 2024b) proposed an ensemble deep CNN integrating MobileNet and Xception with U-Net segmentation for breast cancer detection on ultrasound images, achieving 87.82% accuracy. Abd (Abd El-Hafeez et al. 2025) introduced a novel multiscale attention model achieving 98.08% accuracy and a macro AUC of 0.9978 on binary breast histopathology classification. (Hayum and Daniel Shadrach 2025) combined Cat Swarm Optimization with an enhanced ensemble of EfficientNetB0, ResNet50, and DenseNet121, reaching 98.19% accuracy on breast histopathology images. (Kaddes et al. 2025) proposed a CNN-LSTM hybrid model that captures both spatial and sequential dependencies in mammographic images, achieving 99.90% accuracy. (Guo et al. 2024b) addressed breast cancer clustering in an unsupervised setting using multiscale texture analysis and a dynamic learning strategy, demonstrating the viability of label-efficient approaches for medical imaging.

For skin cancer classification, (Wu et al. 2022) provided a comprehensive review of deep learning methods, highlighting frontier challenges such as data imbalance, domain adaptation, and model robustness. (Yang et al. 2025) proposed a multi-scale attention booster that combines vision transformers and EfficientNet via ensemble majority voting, achieving 95.05% on the ISIC2018 dataset. In lung cancer, (Andrijanova et al. 2025) investigated integrating atomic force microscopy measurements with clinical and histopathological features using Bayesian networks, identifying key predictive features for subtype classification. (Yagappan et al. 2025) proposed the Transformer-based Random Gooseneck Barnacle algorithm, which combines vision transformers with metaheuristic hyperparameter tuning and achieves 99.61% accuracy on lung histopathology images. Multi-task frameworks have also been explored: (Ling et al. 2024) proposed MTANet, a one-stage attention network that simultaneously performs segmentation and clustering across polyp, skin lesion, and liver tumor imaging modalities.

### 2.3 Metaheuristic optimization for image classification

The integration of metaheuristic algorithms into medical image clustering pipelines has been an active research direction. Feature selection is a prominent application: (Mirjalili and Lewis 2016); (Mafarja and Mirjalili 2018) proposed binary variants of the Whale Optimization Algorithm (WOA) for wrapper feature selection, demonstrating superiority over PSO and genetic algorithms. Snow leopard optimization has been applied to high-dimensional genetic data feature selection, outperforming several well-known algorithms in Friedman rankings (Guo et al., 2024a). (Guo et al. 2026) applied DOPSO, a dual oscillatory particle swarm optimizer with anti-phase operators and a dual-subgroup architecture, to tomato disease image classification via LightGBM hyperparameter optimization, achieving 82.25% accuracy. The Gray Wolf Optimizer (GWO) (Mirjalili et al., 2014) simulates the leadership hierarchy and hunting mechanism of gray wolves, using alpha, beta, delta, and omega wolves to guide the population toward the prey (global optimum) through encircling, hunting, and attacking behaviors. The Dung Beetle Optimizer (DBO) (Xue and Shen, 2023) models the ball-rolling, dancing, foraging, stealing, and reproduction behaviors of dung beetles, partitioning the population into role-specific subgroups to jointly pursue global exploration and local exploitation. The Beluga Whale Optimization (BWO) (Zhong et al., 2022) is inspired by the swimming, preying, and whale-fall behaviors of beluga whales, and structures the search process into three explicit phases–exploration, exploitation, and whale fall–to escape local optima.

On the benchmark side, recent metaheuristic proposals have demonstrated strong numerical performance. The Dream Optimization Algorithm incorporates memory retention and dream-sharing strategies, outperforming 27 competing algorithms across the CEC2017, CEC2019, and CEC2022 benchmarks (Lang and Gao, 2025). The Horned Lizard Optimization Algorithm models biological defense strategies including crypsis and blood-squirting, achieving top Friedman rankings on CEC2017 and CEC2019 functions (Peraza-Vázquez et al., 2024). The Snow Ablation Optimizer emulates sublimation and melting behaviors to balance exploration and exploitation, and it has been validated on 29 CEC2017 functions and 22 CEC2020 constrained problems (Deng and Liu, 2023). The Political Optimizer draws on socio-political inspiration and has been benchmarked on a wide range of global optimization problems (Askari et al., 2020).

Despite this diversity of approaches, a systematic gap remains: a substantial portion of metaheuristic algorithms are evaluated exclusively on numerical benchmarks without validation on practical clustering tasks, while a substantial portion of deep learning clustering systems treat hyperparameter tuning as a secondary concern. SMBO is designed to bridge this gap by achieving competitive benchmark performance while serving as an effective optimizer for brain cancer MRI clustering pipelines.

## 3 Materials and methods

### 3.1 Overview

The Sinusoidal Modulation Bi-population Optimizer (SMBO) is a population-based metaheuristic that combines a sinusoidal modulation step size with dynamic behavior switching and asymmetric information flow to balance exploration and exploitation. A heterogeneous population partition (HPP) and an adaptive parameter control (APC) mechanism further support this approach, as detailed below. The algorithm maintains a fixed population of *NP* individuals over *MaxIt* iterations and seeks the global optimum of a *D*-dimensional continuous objective function.

#### 3.1.1 Heterogeneous population partition (HPP)

The population is partitioned into two subgroups of approximately equal size (*N*_*A*_ = ⌊*NP*/2⌋): Group A (Elite Convergence Pool) and Group B (Diversity Exploration Pool). Each group maintains its own local best while sharing the global best position, preventing premature convergence by preserving diversity in Group B while accelerating exploitation in Group A.

#### 3.1.2 Dynamic behavior switching (DBS)

At each iteration, a single behavioral stage is selected probabilistically for the whole population based on the normalized iteration progress *p* = *It*/*MaxIt*, driving the algorithm from broad stochastic exploration toward elite-guided exploitation without any manually tuned transition threshold:


$$\begin{array}{lll} P\left(\text{stage}=1\right) & = & 1-p,\;P\left(\text{stage}=2\right)=p\left(1-p\right), \end{array}$$

$$\begin{array}{lll} P\left(\text{stage}=3\right) & = & p^{2}. \end{array}$$ (1)

Stage 1 dominates the early phase, Stage 2 peaks at *p* = 0.5, and Stage 3 governs late-stage convergence.

#### 3.1.3 Sinusoidal modulation strategy (SMS)

The sinusoidal modulation step size serves as the unified adaptive step size for Stages 2 and 3. In Stage 1, sinusoidal noise replaces the conventional Gaussian perturbation, bounding the perturbation amplitude: ⌈*U*·*D*⌉ dimensions (where U~U(0,1)) are sampled independently with replacement, and each sampled dimension *j* is updated using an independently resampled peer *x*_*r*_, weight *A*_*m*_, direction Flag, and random draw *U*′. The details of the three stage update rules are shown in Equations 2–4:


$$\begin{array}{l} v_{i}^{\left(j\right)}=x_{r}^{\left(j\right)}+\text{Flag}\cdot PR_{i}\cdot A_{m}\cdot \sin\left(2\pi U^{\prime }\right),\;A_{m}=\exp\left(-\frac{f_{i}}{f_{r}+\varepsilon }\right) \end{array}$$ (2)

$$\begin{array}{l} v_{i}=x_{\text{best}}+\Phi \left(p,f_{i}\right)\cdot \left(x_{\text{pbest}}-x_{r_{2}}\right) \end{array}$$ (3)

$$\begin{array}{l} v_{i}=x_{i}+\Phi \left(p,f_{i}\right)\cdot F_{A}\cdot \left(x_{\text{best}}^{\text{other}}-x_{i}\right)+\Phi \left(p,f_{i}\right)\cdot \left(x_{r_{1}}-x_{r_{2}}\right) \end{array}$$ (4)

where Φ(*p, f*_*i*_) =(1 − sin(π*p*/2))·exp(−(*f*_*i*_−*f*_best_)/*f*_range_), with *f*_range_ = *f*_worst_−*f*_best_+ε, is the sinusoidal modulation step size. The first factor enforces a global, monotonic shrinkage of the step size as iterations progress. The second factor couples the step size to each individual's relative fitness quality: since *f*_*i*_≥*f*_best_ always holds, this factor is largest ( → 1) for individuals close to the current global best and decays toward 0 for poorer individuals—i.e., elite individuals are granted larger, more aggressive steps to continue exploiting the promising region around the global best, while below-average individuals receive smaller, conservative corrections. *x*_*r*_ is a randomly selected peer from the same group with fitness *f*_*r*_, Flag∈{+1, −1} is a random direction indicator, $U^{\prime }\sim U\left(0,1\right)$ is an independent random draw (distinct from the dimension-count variable *U* introduced above), *PR*_*i*_ is the perturbation rate of individual *i*, *A*_*m*_ is the fitness-ratio amplitude weight, *x*_pbest_ is drawn from the top elite pool, $x_{\text{best}}^{\text{other}}$ is the best individual of the opposing group, $F_{A}=\exp\left(-f_{\text{best}}^{\text{other}}/\left(f_{i}+\varepsilon \right)\right)$ is the cross-group fitness-ratio guidance weight, and *x*_*r*_1__, *x*_*r*_2__ are randomly selected reference individuals (for Group B, *x*_*r*_2__ is drawn from the AIF archive A instead of the population).

#### 3.1.4 Asymmetric information flow (AIF)

During Stage 3, the worst individual in Group A is ejected into a directed archive A (capped at size *NP*, with random eviction once full), later sampled by Group B as an alternative reference. The second-best individual of Group B is injected into the vacated Group A slot (with a small perturbation) whenever it improves on the ejected individual. This one-directional flow enriches Group B's reference diversity while refreshing Group A's weakest slot with a promising candidate.

#### 3.1.5 Adaptive parameter control (APC)

Two historical memory pools of size *H*, *M*_*SS*_ and *M*_*PR*_, store successful step sizes and perturbation rates. At each iteration, every individual independently draws a memory index $r_{i}\sim U\left\{1,\ldots ,H\right\}$ and samples its parameters from a Cauchy distribution centered on *M*_*SS*_[*r*_*i*_] and a Normal distribution centered on *M*_*PR*_[*r*_*i*_], respectively. After each generation in which at least one individual improves, a single circularly-advancing slot *k* of each pool is updated from its respective set of successful individuals, weighted by fitness improvement (the pools remain unchanged in generations with no successful individuals):


$$\begin{array}{l} M_{PR}\left[k\right]\leftarrow \underset{m}{\sum }w_{m}^{PR}\cdot PR_{m},\;M_{SS}\left[k\right]\leftarrow \frac{\underset{m}{\sum }w_{m}^{SS}\cdot SS_{m}^{2}}{\underset{m}{\sum }w_{m}^{SS}\cdot SS_{m}}, \end{array}$$

$$\begin{array}{l} w_{m}^{\left(\cdot \right)}=\frac{\Delta f_{m}}{\underset{n}{\sum }\Delta f_{n}}, \end{array}$$ (5)

where $\Delta f_{m}=|f_{m}^{\text{old}}-f_{m}^{\text{new}}|$, *PR*_*m*_ ranges over all successful individuals of the generation; *SS*_*m*_ ranges over the (generally smaller) subset of successful individuals from Stages 2–3 only, *k* is the current circularly-advancing memory slot, and *M*_*SS*_[*k*] is updated via the Lehmer mean to bias toward larger successful step sizes.

#### 3.1.6 Pseudo-code of Sinusoidal Modulation Bi-population Optimizer (SMBO)

To provide a clearer introduction to SMBO, the pseudo-code of SMBO is presented in Algorithm 1.

Algorithm 1 Sinusoidal Modulation Bi-population Optimizer (SMBO) 
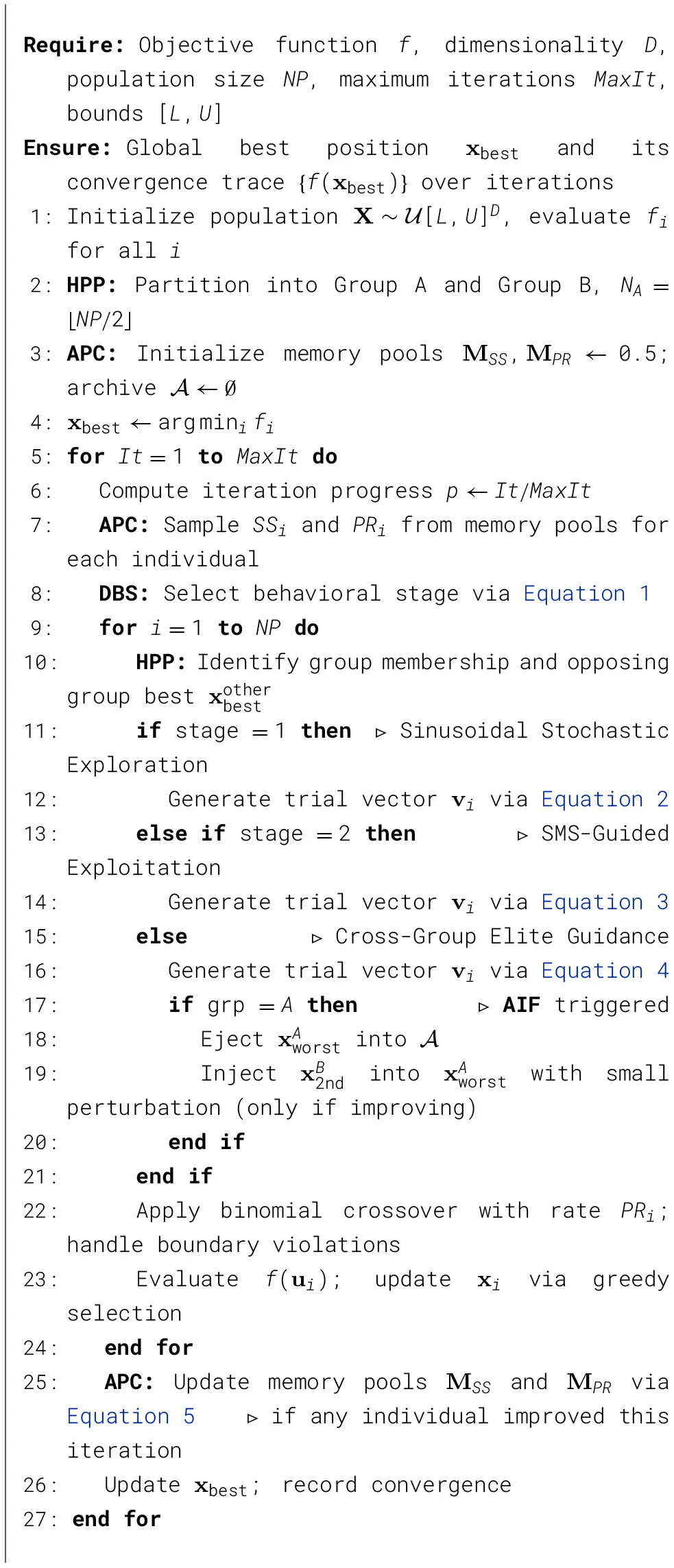

#### 3.1.7 Computational complexity analysis

The per-iteration time complexity of SMBO decomposes into: *O*(*NP*) for HPP group-best identification and APC parameter sampling; *O*(*NP*log*NP*) for sorting the population to obtain the *p*-best pool used in Equation 3; *O*(*NP*·*D*) for trial-vector generation under SMS/AIF (Equations 2–4), binomial crossover, and boundary repair; and *O*(*NP*·*C*_*f*_) for evaluating the *NP* trial vectors, where *C*_*f*_ is the cost of one objective-function evaluation. Summing these terms gives a per-iteration cost of *O*(*NP*·(*D*+log*NP*+*C*_*f*_)), and over *MaxIt* iterations the overall time complexity of SMBO is *O*(*MaxIt*·*NP*·(*D*+log*NP*+*C*_*f*_)), which reduces to the standard *O*(*MaxIt*·*NP*·*D*) of population-based differential-evolution-family metaheuristics when *C*_*f*_ = *O*(*D*).

## 4 Experiments

### 4.1 Experimental results with CEC2017

To evaluate SMBO's search capability across varying problem complexity, we conducted numerical experiments on the CEC2017 benchmark suite at *D* = 10 and *D* = 100. We compared SMBO against five representative metaheuristics (BWO, DBO, DOPSO, GWO, WOA) under identical settings (Pop = 30, 30 independent runs; MaxIt = 10000 at *D* = 10 and MaxIt = 1000 at *D* = 100 to keep the evaluation budget tractable at higher dimensionality). The results show that SMBO achieves the best overall average rank at both dimensionalities (1.38 at *D* = 10 and 1.10 at *D* = 100). DBO is the closest competitor at *D* = 10 (1.90), while DBO and GWO are tied as the closest competitors at *D* = 100 (2.48 each). Experimental results are shown in Tables 1–6. Runtime comparisons are shown in Table 7.

**Table 1:** Comparison of six algorithms on CEC2017 F1–F10 (*D* = 10, Pop = 30, MaxIt = 10, 000, 30 runs), with Wilcoxon rank-sum significance markers relative to SMBO (+, SMBO significantly better; −, SMBO significantly worse; ~, no significant difference; α = 0.05).

Func	Metric	SMBO	BWO	DBO	DOPSO	GWO	WOA
*F*_1_	Mean	**2.49E+03**	4.85E+09	5.61E+03	5.48E+09	4.49E+08	1.12E+09
	Std	1.94E+03	1.42E+09	3.88E+03	1.21E+09	1.49E+08	1.08E+09
	Rank	**1**	5	2	6	3	4
	Sig.	–	+	+	+	+	+
*F*_2_	Mean	1.22E-07	6.54E+03	**1.74E-13**	3.14E+04	5.25E+03	8.25E+03
	Std	1.15E-07	1.17E+03	3.36E-13	5.80E+03	1.92E+03	3.51E+03
	Rank	2	4	**1**	6	3	5
	Sig.	–	+	−	+	+	+
*F*_3_	Mean	**1.75E-01**	1.95E+02	5.34E+00	3.85E+02	2.57E+01	1.09E+02
	Std	1.27E-01	7.47E+01	3.42E+00	1.10E+02	9.75E+00	8.93E+01
	Rank	**1**	5	2	6	3	4
	Sig.	–	+	+	+	+	+
*F*_4_	Mean	**1.37E+01**	6.91E+01	2.60E+01	8.72E+01	3.67E+01	5.40E+01
	Std	5.12E+00	8.73E+00	1.09E+01	9.07E+00	4.23E+00	2.01E+01
	Rank	**1**	5	2	6	3	4
	Sig.	–	+	+	+	+	+
*F*_5_	Mean	**8.44E-07**	3.56E+01	4.47E+00	5.53E+01	1.13E+01	2.85E+01
	Std	2.26E-06	4.99E+00	4.17E+00	6.48E+00	2.45E+00	1.06E+01
	Rank	**1**	5	2	6	3	4
	Sig.	–	+	+	+	+	+
*F*_6_	Mean	**3.38E+01**	8.65E+01	4.35E+01	2.90E+02	5.68E+01	8.30E+01
	Std	9.24E+00	7.08E+00	1.42E+01	5.33E+01	4.24E+00	2.24E+01
	Rank	**1**	5	2	6	3	4
	Sig.	–	+	+	+	+	+
*F*_7_	Mean	**1.49E+01**	4.37E+01	2.50E+01	1.00E+02	2.90E+01	3.82E+01
	Std	5.42E+00	3.66E+00	9.41E+00	1.15E+01	5.23E+00	1.15E+01
	Rank	**1**	5	2	6	3	4
	Sig.	–	+	+	+	+	+
*F*_8_	Mean	**2.76E+00**	3.69E+02	3.98E+01	2.36E+03	5.52E+01	4.17E+02
	Std	1.45E+01	8.50E+01	8.88E+01	6.19E+02	1.74E+01	2.39E+02
	Rank	**1**	4	2	6	3	5
	Sig.	–	+	+	+	+	+
*F*_9_	Mean	**3.76E+02**	1.17E+03	7.39E+02	9.32E+02	8.09E+02	1.23E+03
	Std	2.15E+02	1.80E+02	2.47E+02	2.08E+02	3.01E+02	3.28E+02
	Rank	**1**	5	2	4	3	6
	Sig.	–	+	+	+	+	+
*F*_10_	Mean	**1.48E+01**	4.23E+02	5.29E+01	1.34E+03	1.12E+02	2.94E+02
	Std	9.89E+00	2.02E+02	4.18E+01	8.43E+02	1.18E+02	4.24E+02
	Rank	**1**	5	2	6	3	4
	Sig.	–	+	+	+	+	+

Bold: best Mean or Rank = 1.

**Table 2:** Comparison of six algorithms on CEC2017 F11–F20 (*D* = 10, Pop = 30, MaxIt = 10, 000, 30 runs), with Wilcoxon rank-sum significance markers relative to SMBO (+, SMBO significantly better; −, SMBO significantly worse; ~, no significant difference; α = 0.05).

Func	Metric	SMBO	BWO	DBO	DOPSO	GWO	WOA
*F*_11_	Mean	**1.60E+04**	1.62E+07	1.32E+06	2.25E+08	1.83E+06	5.67E+06
	Std	1.36E+04	9.85E+06	4.52E+06	9.68E+07	1.64E+06	6.63E+06
	Rank	**1**	5	2	6	3	4
	Sig.	–	+	~	+	+	+
*F*_12_	Mean	**3.69E+01**	1.39E+05	5.93E+03	2.90E+05	7.46E+03	1.13E+04
	Std	7.35E+01	1.09E+05	9.81E+03	4.77E+05	4.32E+02	7.44E+03
	Rank	**1**	5	2	6	3	4
	Sig.	–	+	+	+	+	+
*F*_13_	Mean	**1.11E+01**	1.44E+02	9.00E+01	6.15E+02	1.58E+03	1.60E+02
	Std	9.50E+00	2.57E+01	4.79E+01	3.92E+02	1.32E+03	5.40E+01
	Rank	**1**	3	2	5	6	4
	Sig.	–	+	+	+	+	+
*F*_14_	Mean	**7.09E+00**	3.03E+03	1.41E+02	3.71E+03	3.28E+03	7.21E+03
	Std	6.88E+00	1.50E+03	1.15E+02	1.90E+03	1.56E+03	2.76E+03
	Rank	**1**	3	2	5	4	6
	Sig.	–	+	+	+	+	+
*F*_15_	Mean	**1.09E+02**	2.51E+02	1.20E+02	2.67E+02	1.34E+02	3.11E+02
	Std	9.41E+01	8.37E+01	1.15E+02	1.83E+02	8.14E+01	1.30E+02
	Rank	**1**	4	2	5	3	6
	Sig.	–	+	~	+	~	+
*F*_16_	Mean	6.17E+01	8.91E+01	**5.50E+01**	1.59E+02	7.55E+01	9.71E+01
	Std	5.20E+01	1.09E+01	1.62E+01	3.07E+01	8.02E+00	4.34E+01
	Rank	2	4	**1**	6	3	5
	Sig.	–	+	~	+	~	+
*F*_17_	Mean	**1.26E+02**	2.60E+05	1.54E+04	1.02E+05	2.38E+04	1.22E+04
	Std	4.21E+02	3.51E+05	1.81E+04	8.02E+04	2.73E+03	9.13E+03
	Rank	**1**	6	3	5	4	2
	Sig.	–	+	+	+	+	+
*F*_18_	Mean	**4.17E+00**	1.30E+04	1.52E+02	7.03E+04	1.41E+04	1.83E+04
	Std	3.84E+00	9.70E+03	2.38E+02	4.19E+04	9.06E+03	3.90E+04
	Rank	**1**	3	2	6	4	5
	Sig.	–	+	+	+	+	+
*F*_19_	Mean	**2.69E+01**	1.55E+02	7.11E+01	2.44E+02	1.36E+02	1.79E+02
	Std	3.31E+01	3.90E+01	5.26E+01	5.68E+01	3.17E+01	5.51E+01
	Rank	**1**	4	2	6	3	5
	Sig.	–	+	+	+	+	+
*F*_20_	Mean	2.02E+02	1.42E+02	**1.03E+02**	1.49E+02	2.34E+02	2.32E+02
	Std	4.03E+01	1.18E+01	1.52E+00	1.72E+01	4.53E+00	4.10E+01
	Rank	4	2	**1**	3	6	5
	Sig.	–	−	−	−	+	+

Bold: best Mean or Rank = 1.

**Table 3:** Comparison of six algorithms on CEC2017 F21–F29 (*D* = 10, Pop = 30, MaxIt = 10, 000, 30 runs), with Wilcoxon rank-sum significance markers relative to SMBO (+, SMBO significantly better; −, SMBO significantly worse; ~, no significant difference; α = 0.05).

Func	Metric	SMBO	BWO	DBO	DOPSO	GWO	WOA
*F*_21_	Mean	**9.74E+01**	2.21E+02	1.06E+02	7.46E+02	1.33E+02	2.47E+02
	Std	1.74E+01	8.50E+01	1.24E+01	2.54E+02	5.01E+00	2.56E+02
	Rank	**1**	4	2	6	3	5
	Sig.	–	+	+	+	+	+
*F*_22_	Mean	**3.17E+02**	3.68E+02	3.34E+02	3.76E+02	3.41E+02	3.57E+02
	Std	6.60E+00	1.23E+01	1.37E+01	1.42E+01	4.31E+00	2.83E+01
	Rank	**1**	5	2	6	3	4
	Sig.	–	+	+	+	+	+
*F*_23_	Mean	3.49E+02	2.55E+02	**1.77E+02**	4.14E+02	3.65E+02	4.02E+02
	Std	6.71E+00	5.61E+01	1.12E+02	1.62E+01	5.64E+00	5.10E+01
	Rank	3	2	**1**	6	4	5
	Sig.	–	−	−	+	+	+
*F*_24_	Mean	4.35E+02	5.91E+02	**4.09E+02**	7.57E+02	4.35E+02	5.25E+02
	Std	2.00E+01	4.22E+01	8.64E+01	8.11E+01	7.07E+00	1.07E+02
	Rank	3	5	**1**	6	2	4
	Sig.	–	+	~	+	−	+
*F*_25_	Mean	**3.94E+02**	8.51E+02	4.37E+02	9.33E+02	4.64E+02	1.00E+03
	Std	1.17E+02	1.35E+02	1.18E+02	3.84E+02	1.87E+02	5.21E+02
	Rank	**1**	4	2	5	3	6
	Sig.	–	+	+	+	+	+
*F*_26_	Mean	4.03E+02	4.27E+02	3.97E+02	4.13E+02	**3.96E+02**	4.66E+02
	Std	7.00E+00	8.08E+00	3.10E+00	9.28E+00	1.03E+00	4.48E+01
	Rank	3	5	2	4	**1**	6
	Sig.	–	+	−	+	−	+
*F*_27_	Mean	**4.32E+02**	6.71E+02	4.98E+02	6.40E+02	5.56E+02	7.13E+02
	Std	1.37E+02	1.04E+02	1.19E+02	1.27E+02	8.08E+01	2.20E+02
	Rank	**1**	5	2	4	3	6
	Sig.	–	+	+	+	+	+
*F*_28_	Mean	**2.79E+02**	4.00E+02	3.11E+02	3.69E+02	3.20E+02	4.39E+02
	Std	2.90E+01	3.14E+01	6.20E+01	3.92E+01	1.85E+01	7.82E+01
	Rank	**1**	5	2	4	3	6
	Sig.	–	+	~	+	+	+
*F*_29_	Mean	**1.72E+05**	1.03E+06	7.41E+05	1.16E+06	4.79E+05	3.17E+06
	Std	4.21E+05	5.00E+05	1.29E+06	8.59E+05	7.92E+05	5.36E+06
	Rank	**1**	4	3	5	2	6
	Sig.	–	+	~	+	+	+
Avg Rank		1.38	4.34	1.90	5.41	3.21	4.76
SMBO W/T/L		–	27/0/2	19/6/4	28/0/1	25/2/2	29/0/0

Bold: best Mean or Rank = 1.

**Table 4:** Comparison of six algorithms on CEC2017 F1–F10 (*D* = 100, Pop = 30, MaxIt = 1, 000, 30 runs), with Wilcoxon rank-sum significance markers relative to SMBO (+, SMBO significantly better; −, SMBO significantly worse; ~, no significant difference; α = 0.05).

Func	Metric	SMBO	BWO	DBO	DOPSO	GWO	WOA
*F*_1_	Mean	**2.35E+07**	2.57E+11	4.90E+10	5.72E+11	7.96E+10	2.37E+11
	Std	1.13E+07	6.92E+09	5.93E+10	4.42E+10	1.25E+10	1.65E+10
	Rank	**1**	5	2	6	3	4
	Sig.	–	+	+	+	+	+
*F*_2_	Mean	6.51E+05	3.60E+05	5.42E+05	1.28E+06	**3.55E+05**	7.37E+05
	Std	5.85E+04	2.06E+04	2.15E+05	2.41E+05	1.87E+04	2.60E+05
	Rank	4	2	3	6	**1**	5
	Sig.	–	−	−	+	−	~
*F*_3_	Mean	**5.42E+02**	9.51E+04	1.63E+04	2.52E+05	8.88E+03	7.70E+04
	Std	9.47E+01	6.20E+03	1.64E+04	4.72E+04	1.85E+03	1.30E+04
	Rank	**1**	5	3	6	2	4
	Sig.	–	+	+	+	+	+
*F*_4_	Mean	**5.22E+02**	1.61E+03	1.05E+03	2.52E+03	1.15E+03	1.60E+03
	Std	1.23E+02	2.72E+01	2.35E+02	1.39E+02	5.15E+01	5.67E+01
	Rank	**1**	5	2	6	3	4
	Sig.	–	+	+	+	+	+
*F*_5_	Mean	**1.82E+01**	1.11E+02	7.35E+01	1.58E+02	7.22E+01	1.10E+02
	Std	4.71E+00	2.48E+00	1.15E+01	9.10E+00	4.02E+00	5.28E+00
	Rank	**1**	5	3	6	2	4
	Sig.	–	+	+	+	+	+
*F*_6_	Mean	**1.07E+03**	3.19E+03	2.12E+03	1.21E+04	1.81E+03	3.30E+03
	Std	2.18E+02	5.12E+01	2.71E+02	9.15E+02	1.01E+02	1.00E+02
	Rank	**1**	4	3	6	2	5
	Sig.	–	+	+	+	+	+
*F*_7_	Mean	**4.85E+02**	1.78E+03	1.25E+03	2.66E+03	1.16E+03	1.75E+03
	Std	1.56E+02	3.85E+01	2.13E+02	1.98E+02	5.30E+01	8.68E+01
	Rank	**1**	5	3	6	2	4
	Sig.	–	+	+	+	+	+
*F*_8_	Mean	**1.65E+04**	7.73E+04	6.76E+04	2.05E+05	5.20E+04	7.41E+04
	Std	6.85E+03	3.54E+03	1.57E+04	2.00E+04	3.27E+03	5.78E+03
	Rank	**1**	5	3	6	2	4
	Sig.	–	+	+	+	+	+
*F*_9_	Mean	**1.92E+04**	3.12E+04	2.43E+04	3.19E+04	2.49E+04	2.96E+04
	Std	9.89E+02	5.94E+02	5.14E+03	7.52E+02	1.26E+03	1.51E+03
	Rank	**1**	5	2	6	3	4
	Sig.	–	+	+	+	+	+
*F*_10_	Mean	**6.35E+04**	3.05E+05	1.63E+05	6.45E+05	1.80E+05	4.07E+05
	Std	2.00E+04	4.52E+04	3.57E+04	2.00E+05	2.23E+04	1.71E+05
	Rank	**1**	4	2	6	3	5
	Sig.	–	+	+	+	+	+

Bold: best Mean or Rank = 1.

**Table 5:** Comparison of six algorithms on CEC2017 F11–F20 (*D* = 100, Pop = 30, MaxIt = 1, 000, 30 runs), with Wilcoxon rank-sum significance markers relative to SMBO (+, SMBO significantly better; −, SMBO significantly worse; ~, no significant difference; α = 0.05).

Func	Metric	SMBO	BWO	DBO	DOPSO	GWO	WOA
*F*_11_	Mean	**1.56E+08**	1.89E+11	3.28E+09	2.89E+11	2.18E+10	1.45E+11
	Std	7.79E+07	9.88E+09	1.23E+09	2.41E+10	4.44E+09	2.45E+10
	Rank	**1**	5	2	6	3	4
	Sig.	–	+	+	+	+	+
*F*_12_	Mean	**5.58E+04**	4.24E+10	1.52E+08	6.74E+10	3.68E+09	3.20E+10
	Std	2.26E+04	3.00E+09	1.55E+08	1.03E+10	1.01E+09	6.95E+09
	Rank	**1**	5	2	6	3	4
	Sig.	–	+	+	+	+	+
*F*_13_	Mean	**4.22E+06**	7.47E+07	1.65E+07	1.80E+08	1.83E+07	4.70E+07
	Std	3.85E+06	1.86E+07	1.12E+07	5.45E+07	6.49E+06	2.18E+07
	Rank	**1**	5	2	6	3	4
	Sig.	–	+	+	+	+	+
*F*_14_	Mean	**2.30E+04**	2.14E+10	3.99E+07	2.63E+10	1.02E+09	1.20E+10
	Std	7.80E+03	2.60E+09	6.35E+07	6.26E+09	9.71E+08	3.56E+09
	Rank	**1**	5	2	6	3	4
	Sig.	–	+	+	+	+	+
*F*_15_	Mean	**4.61E+03**	2.04E+04	7.52E+03	2.01E+04	8.70E+03	1.99E+04
	Std	4.76E+02	1.38E+03	1.59E+03	1.77E+03	7.29E+02	2.66E+03
	Rank	**1**	6	2	5	3	4
	Sig.	–	+	+	+	+	+
*F*_16_	Mean	**3.24E+03**	3.51E+06	6.92E+03	8.64E+06	7.53E+03	1.29E+06
	Std	6.01E+02	1.74E+06	1.46E+03	4.71E+06	1.50E+03	1.45E+06
	Rank	**1**	5	2	6	3	4
	Sig.	–	+	+	+	+	+
*F*_17_	Mean	**8.45E+06**	1.94E+08	1.64E+07	2.57E+08	2.35E+07	5.74E+07
	Std	4.88E+06	5.56E+07	8.73E+06	9.46E+07	1.00E+07	2.89E+07
	Rank	**1**	5	2	6	3	4
	Sig.	–	+	+	+	+	+
*F*_18_	Mean	**4.55E+04**	2.14E+10	3.71E+07	2.75E+10	1.01E+09	1.07E+10
	Std	4.75E+04	2.63E+09	2.65E+07	3.54E+09	5.79E+08	3.91E+09
	Rank	**1**	5	2	6	3	4
	Sig.	–	+	+	+	+	+
*F*_19_	Mean	**2.90E+03**	5.67E+03	4.65E+03	6.63E+03	4.59E+03	5.58E+03
	Std	2.82E+02	2.14E+02	8.93E+02	2.61E+02	3.81E+02	4.63E+02
	Rank	**1**	5	3	6	2	4
	Sig.	–	+	+	+	+	+
*F*_20_	Mean	**7.69E+02**	2.63E+03	1.88E+03	3.16E+03	1.33E+03	2.63E+03
	Std	1.12E+02	7.80E+01	1.92E+02	1.57E+02	8.59E+01	2.41E+02
	Rank	**1**	4	3	6	2	5
	Sig.	–	+	+	+	+	+

Bold: best Mean or Rank = 1.

**Table 6:** Comparison of six algorithms on CEC2017 F21–F29 (*D* = 100, Pop = 30, MaxIt = 1, 000, 30 runs), with Wilcoxon rank-sum significance markers relative to SMBO (+, SMBO significantly better; −, SMBO significantly worse; ~, no significant difference; α = 0.05).

Func	Metric	SMBO	BWO	DBO	DOPSO	GWO	WOA
*F*_21_	Mean	**2.07E+04**	3.23E+04	2.65E+04	3.22E+04	2.59E+04	3.17E+04
	Std	6.35E+02	7.42E+02	5.50E+03	1.09E+03	3.55E+03	1.06E+03
	Rank	**1**	6	3	5	2	4
	Sig.	–	+	+	+	+	+
*F*_22_	Mean	**9.86E+02**	3.76E+03	2.42E+03	2.79E+03	1.82E+03	3.95E+03
	Std	8.05E+01	1.24E+02	2.14E+02	1.70E+02	1.10E+02	4.27E+02
	Rank	**1**	5	3	4	2	6
	Sig.	–	+	+	+	+	+
*F*_23_	Mean	**1.49E+03**	6.64E+03	3.67E+03	3.86E+03	2.42E+03	6.89E+03
	Std	1.16E+02	3.12E+02	5.61E+02	3.05E+02	2.06E+02	1.15E+03
	Rank	**1**	5	3	4	2	6
	Sig.	–	+	+	+	+	+
*F*_24_	Mean	**1.18E+03**	2.47E+04	5.92E+03	1.20E+05	5.87E+03	2.04E+04
	Std	8.51E+01	1.25E+03	5.98E+03	1.94E+04	1.01E+03	2.00E+03
	Rank	**1**	5	3	6	2	4
	Sig.	–	+	+	+	+	+
*F*_25_	Mean	**9.16E+03**	4.80E+04	2.08E+04	3.32E+04	1.92E+04	4.60E+04
	Std	1.21E+03	1.47E+03	3.14E+03	3.01E+03	1.09E+03	4.34E+03
	Rank	**1**	6	3	4	2	5
	Sig.	–	+	+	+	+	+
*F*_26_	Mean	**1.25E+03**	9.07E+03	1.98E+03	2.94E+03	2.53E+03	7.83E+03
	Std	1.06E+02	9.22E+02	4.53E+02	5.05E+02	2.35E+02	1.98E+03
	Rank	**1**	6	2	4	3	5
	Sig.	–	+	+	+	+	+
*F*_27_	Mean	**1.55E+03**	2.45E+04	1.44E+04	2.85E+04	7.77E+03	2.38E+04
	Std	4.10E+02	6.31E+02	7.09E+03	5.61E+03	1.49E+03	1.59E+03
	Rank	**1**	5	3	6	2	4
	Sig.	–	+	+	+	+	+
*F*_28_	Mean	**4.53E+03**	3.74E+05	8.19E+03	3.40E+06	1.08E+04	1.12E+05
	Std	6.47E+02	1.55E+05	2.01E+03	1.91E+06	1.14E+03	1.01E+05
	Rank	**1**	5	2	6	3	4
	Sig.	–	+	+	+	+	+
*F*_29_	Mean	**8.20E+06**	3.85E+10	1.17E+08	4.10E+10	2.90E+09	2.25E+10
	Std	3.70E+06	4.13E+09	6.81E+07	6.36E+09	1.16E+09	8.19E+09
	Rank	**1**	5	2	6	3	4
	Sig.	–	+	+	+	+	+
Avg Rank		1.10	4.93	2.48	5.66	2.48	4.34
SMBO W/T/L		–	28/0/1	28/0/1	29/0/0	28/0/1	28/1/0

Bold: best Mean or Rank = 1.

**Table 7:** Per-function mean wall-clock time (seconds, over 30 runs) for the six compared algorithms on CEC2017 F1–F29 (*D* = 10, Pop = 30, MaxIt = 10, 000).

Func	SMBO	BWO	DBO	DOPSO	GWO	WOA
*F*_1_	11.08	21.97	15.39	14.78	**5.66**	6.41
*F*_2_	10.82	23.05	15.32	14.26	**5.59**	6.17
*F*_3_	10.61	22.80	15.22	14.53	**5.44**	6.10
*F*_4_	10.82	22.52	15.52	14.64	**5.49**	6.54
*F*_5_	11.02	24.55	16.98	15.23	**6.35**	6.74
*F*_6_	11.36	23.59	15.86	14.94	**5.72**	6.32
*F*_7_	11.43	22.54	15.66	14.46	**5.55**	6.53
*F*_8_	11.50	23.60	15.86	14.77	**5.73**	6.30
*F*_9_	11.56	22.53	16.07	14.61	**5.67**	6.61
*F*_10_	10.63	20.77	15.38	14.63	**5.68**	6.07
*F*_11_	10.76	21.16	15.69	14.89	**5.58**	6.21
*F*_12_	11.59	20.94	15.74	14.88	**5.76**	6.53
*F*_13_	11.54	20.76	16.03	14.80	**5.83**	6.40
*F*_14_	11.37	20.50	15.31	14.24	**5.40**	6.33
*F*_15_	11.35	20.41	15.61	14.62	**5.54**	6.25
*F*_16_	10.41	21.10	16.41	14.93	**6.07**	6.43
*F*_17_	10.14	22.68	15.83	14.55	**5.61**	6.43
*F*_18_	11.69	23.77	19.18	15.83	**7.42**	7.70
*F*_19_	10.72	23.64	16.46	14.82	**5.92**	6.67
*F*_20_	10.89	24.22	17.09	15.18	**6.20**	6.79
*F*_21_	10.82	23.57	17.41	15.49	**6.54**	7.12
*F*_22_	10.85	23.93	17.55	15.66	**6.48**	6.99
*F*_23_	11.37	24.23	18.35	15.79	**6.64**	7.41
*F*_24_	10.78	23.36	17.52	15.38	**6.45**	7.05
*F*_25_	11.26	24.20	18.31	16.13	**6.73**	7.42
*F*_26_	11.47	22.90	18.59	15.83	**7.05**	7.64
*F*_27_	11.45	21.70	17.94	15.42	**6.56**	7.14
*F*_28_	10.76	21.62	17.78	15.69	**6.69**	7.27
*F*_29_	12.20	23.02	20.42	16.92	**7.80**	8.24
Average	11.11	22.61	16.71	15.10	6.11	6.75

Bold: fastest per function.

On the CEC2017 benchmark at *D* = 10 (Pop = 30, MaxIt = 10, 000, 30 independent runs), SMBO achieved the best overall average rank (1.38) among the six algorithms, outperforming DBO (1.90), GWO (3.21), BWO (4.34), WOA (4.76), and DOPSO (5.41). A Friedman test across all 29 functions confirmed that these rank differences are highly significant (χ^2^ = 108.67, *p* < 0.001), rejecting the null hypothesis that the six algorithms perform equivalently. Pairwise Wilcoxon rank-sum tests (SMBO vs. each baseline, α = 0.05) further showed that SMBO significantly outperformed WOA on all 29 functions (29/0/0 win/tie/loss), DOPSO on 28 of 29, BWO on 27 of 29, and GWO on 25 of 29. Together, these results indicate that, on the standard 10-dimensional CEC2017 suite—spanning unimodal, simple multimodal, hybrid, and composition landscapes—SMBO's advantage over baselines is consistent and statistically robust. Convergence curves are shown in Figures 1–5.

**Figure 1:**
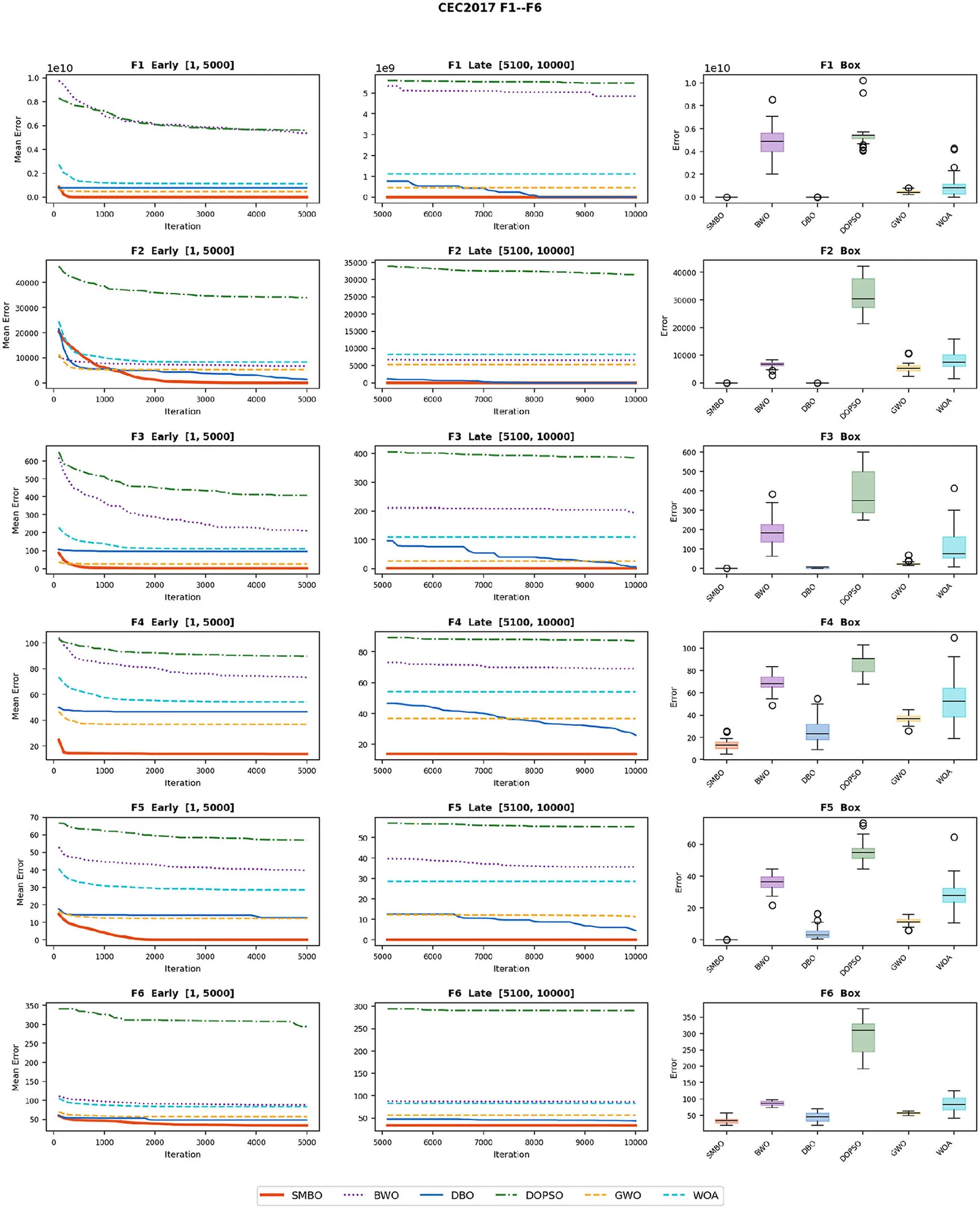
Convergence curves and error distributions of six algorithms on CEC2017 benchmark functions F1–F6.

**Figure 2:**
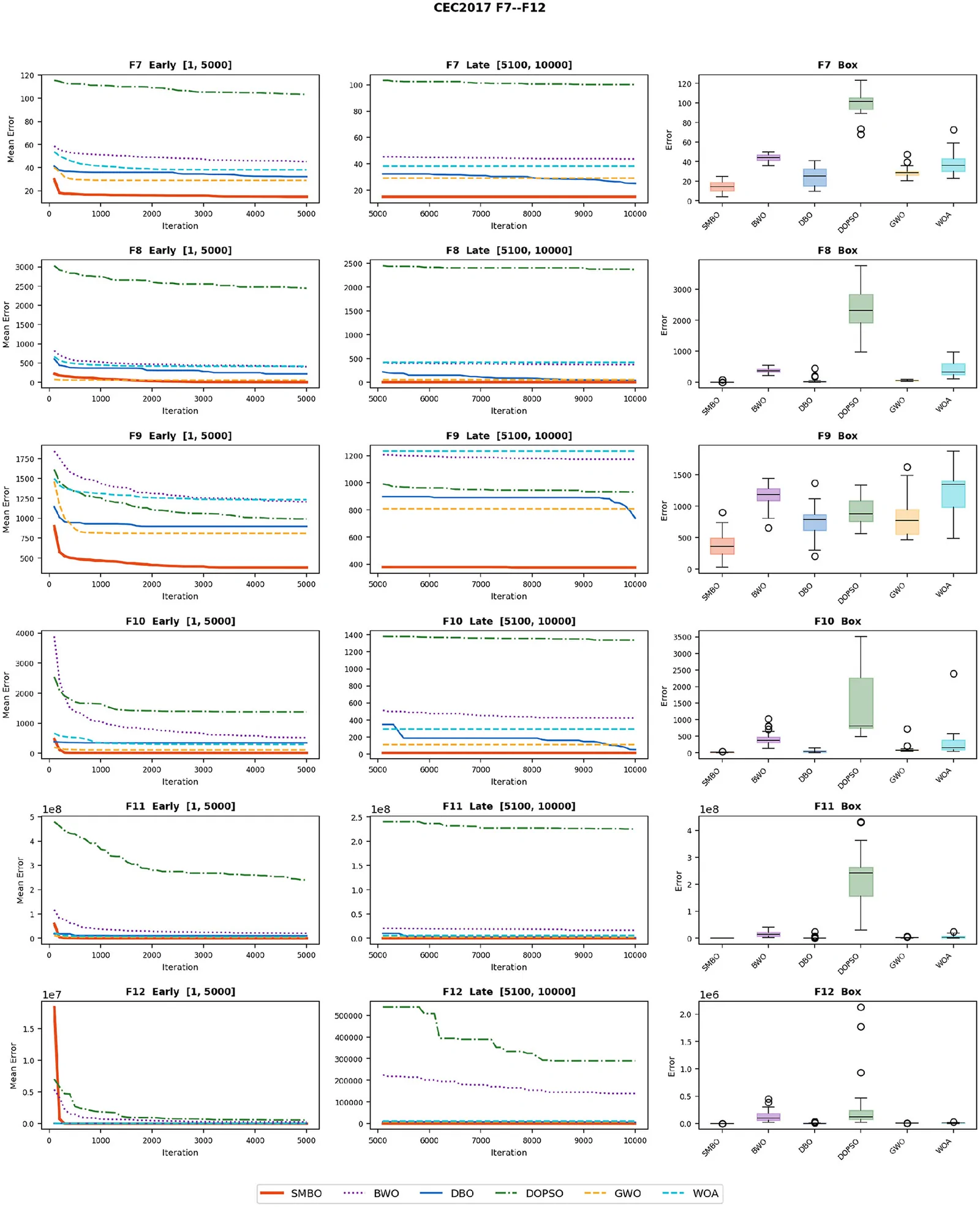
Convergence curves and error distributions of six algorithms on CEC2017 benchmark functions F7–F12.

On the CEC2017 benchmark at *D* = 100 (Pop = 30, MaxIt = 1, 000, 30 independent runs), SMBO achieved the lowest average rank among the six algorithms (1.10), with DBO and GWO tied for the next-best rank (2.48 each), followed by WOA (4.34), BWO (4.93), and DOPSO (5.66). A Friedman test across all 29 functions showed statistically significant differences in average rank among the six algorithms. These results indicate that SMBO maintains stable search performance in high-dimensional environments.

### 4.2 Experimental results with CEC2022

On the CEC2022 benchmark (*D* = 10, Pop = 30, MaxIt = 10, 000, 30 independent runs), SMBO achieved the lowest average rank among the six algorithms (1.33), followed by DBO (1.75), GWO (3.25), BWO and WOA (4.58 each), and DOPSO (5.50). A Friedman test across all 12 functions indicated statistically significant differences in average rank among the six algorithms (χ^2^ = 48.57, *p* < 0.001). Pairwise Wilcoxon rank-sum tests (SMBO vs. each baseline) showed that SMBO's mean error was significantly lower on all 12 functions compared with BWO, DOPSO, and WOA, and on 11 of 12 functions compared with GWO. Tables 8, 9 report the numerical results. Convergence curves are shown in Figures 6, 7.

**Table 8:** Comparison of six algorithms on CEC2022 F1–F6 (*D* = 10, Pop = 30, MaxIt = 10, 000, 30 runs), with Wilcoxon rank-sum significance markers relative to SMBO (+, SMBO significantly better; −, SMBO significantly worse; ~, no significant difference; α = 0.05).

Func	Metric	SMBO	BWO	DBO	DOPSO	GWO	WOA
*F*_1_	Mean	9.96E-08	3.83E+03	**6.06E-14**	2.35E+04	6.06E+03	8.05E+03
	Std	1.12E-07	9.89E+02	6.39E-14	5.65E+03	2.66E+03	2.01E+03
	Rank	2	3	**1**	6	4	5
	Sig.	–	+	−	+	+	+
*F*_2_	Mean	**6.08E+00**	1.89E+02	1.51E+01	2.79E+02	4.34E+01	1.63E+02
	Std	1.26E+01	9.05E+01	2.41E+01	1.16E+02	1.06E+01	1.43E+02
	Rank	**1**	5	2	6	3	4
	Sig.	–	+	+	+	+	+
*F*_3_	Mean	**2.82E-07**	3.53E+01	5.69E+00	5.72E+01	1.11E+01	2.84E+01
	Std	6.28E-07	4.90E+00	4.95E+00	8.97E+00	2.45E+00	1.25E+01
	Rank	**1**	5	2	6	3	4
	Sig.	–	+	+	+	+	+
*F*_4_	Mean	**1.77E+01**	3.92E+01	2.70E+01	8.81E+01	3.08E+01	3.54E+01
	Std	6.15E+00	3.25E+00	7.74E+00	1.18E+01	6.60E+00	8.52E+00
	Rank	**1**	5	2	6	3	4
	Sig.	–	+	+	+	+	+
*F*_5_	Mean	**2.50E+01**	3.54E+02	3.13E+01	2.18E+03	4.20E+01	3.51E+02
	Std	7.22E+01	8.11E+01	2.69E+01	4.04E+02	1.56E+01	2.01E+02
	Rank	**1**	5	2	6	3	4
	Sig.	–	+	+	+	+	+
*F*_6_	Mean	**1.16E+03**	2.56E+05	2.97E+03	6.47E+06	1.07E+05	4.30E+05
	Std	1.46E+03	2.22E+05	2.51E+03	4.26E+06	2.43E+05	1.60E+06
	Rank	**1**	4	2	6	3	5
	Sig.	–	+	+	+	+	+

Bold: best Mean or Rank = 1.

**Table 9:** Comparison of six algorithms on CEC2022 F7–F12 (*D* = 10, Pop = 30, MaxIt = 10, 000, 30 runs), with Wilcoxon rank-sum significance markers relative to SMBO (+, SMBO significantly better; −, SMBO significantly worse; ~, no significant difference; α = 0.05).

Func	Metric	SMBO	BWO	DBO	DOPSO	GWO	WOA
*F*_7_	Mean	**1.60E+01**	7.49E+01	2.83E+01	1.08E+02	5.44E+01	6.82E+01
	Std	1.70E+01	1.09E+01	8.34E+00	1.01E+01	5.85E+00	3.11E+01
	Rank	**1**	5	2	6	3	4
	Sig.	–	+	+	+	+	+
*F*_8_	Mean	**1.89E+01**	3.25E+01	2.41E+01	4.43E+01	3.13E+01	3.09E+01
	Std	4.94E+00	2.11E+00	5.96E+00	3.60E+00	1.74E+00	1.18E+01
	Rank	**1**	5	2	6	4	3
	Sig.	–	+	+	+	+	+
*F*_9_	Mean	**2.29E+02**	3.60E+02	2.29E+02	3.42E+02	2.75E+02	3.36E+02
	Std	3.56E-07	2.12E+01	2.53E-01	6.76E+01	1.97E+01	5.53E+01
	Rank	**1**	6	2	5	3	4
	Sig.	–	+	~	+	+	+
*F*_10_	Mean	1.10E+02	1.25E+02	**1.08E+02**	1.41E+02	1.84E+02	2.75E+02
	Std	5.62E+01	2.53E+01	2.88E+01	1.95E+01	6.34E+01	2.37E+02
	Rank	2	3	**1**	4	5	6
	Sig.	–	+	−	+	+	+
*F*_11_	Mean	**7.36E+01**	3.80E+02	7.71E+01	4.17E+02	3.34E+02	4.19E+02
	Std	1.02E+02	8.34E+01	1.10E+02	1.03E+02	2.05E+02	2.91E+02
	Rank	**1**	4	2	5	3	6
	Sig.	–	+	+	+	+	+
*F*_12_	Mean	1.70E+02	1.84E+02	**1.66E+02**	1.75E+02	1.66E+02	2.40E+02
	Std	5.89E+00	7.20E+00	2.97E+00	6.43E+00	2.94E+00	4.69E+01
	Rank	3	5	**1**	4	2	6
	Sig.	–	+	−	+	−	+
Avg Rank		1.33	4.58	1.75	5.50	3.25	4.58
SMBO W/T/L		–	12/0/0	8/1/3	12/0/0	11/0/1	12/0/0

Bold: best Mean or Rank = 1.

Friedman test (all F1–F12): χ^2^ = 48.57, *p* < 0.001 (df = 5).

**Figure 3:**
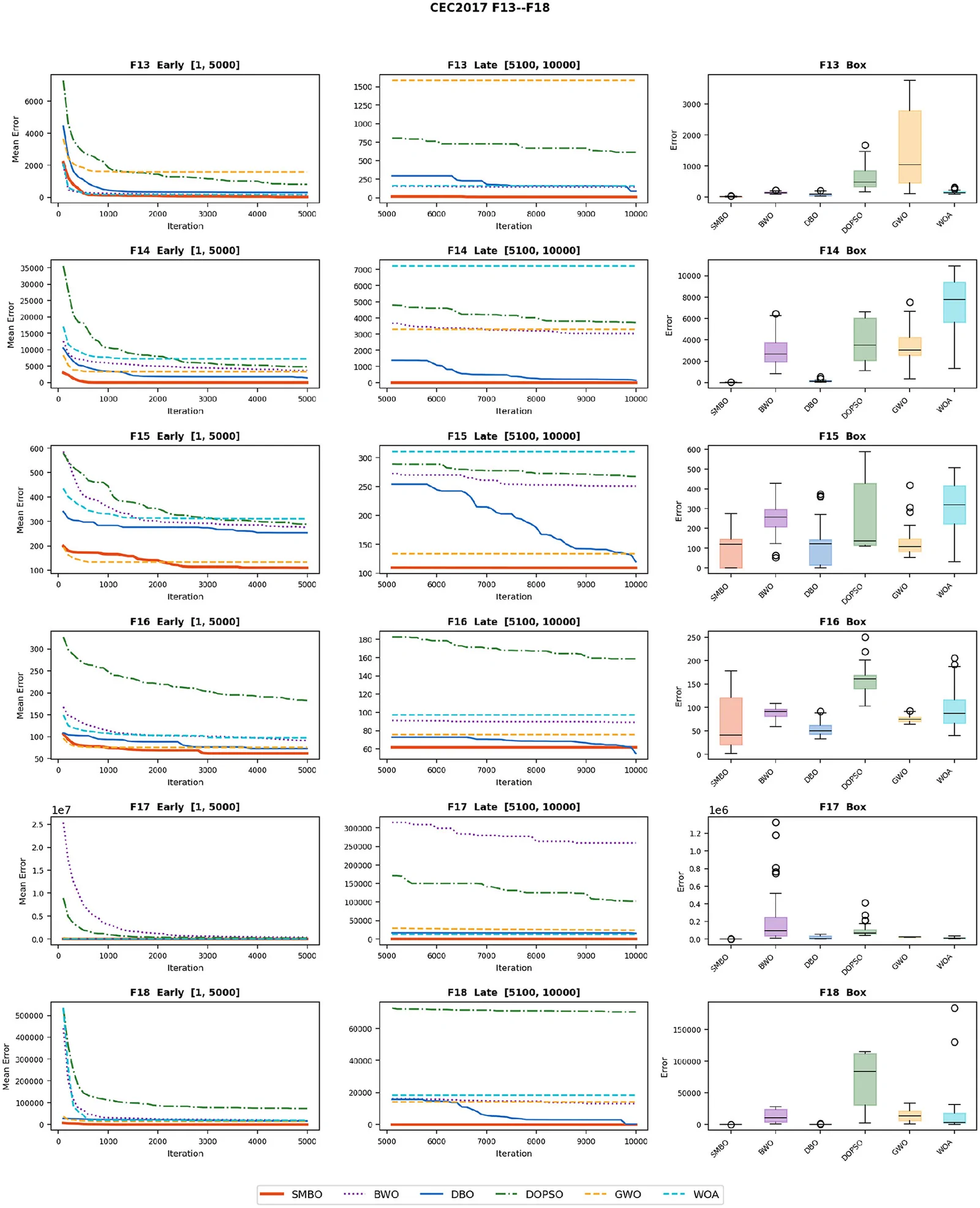
Convergence curves and error distributions of six algorithms on CEC2017 benchmark functions F13–F18.

**Figure 4:**
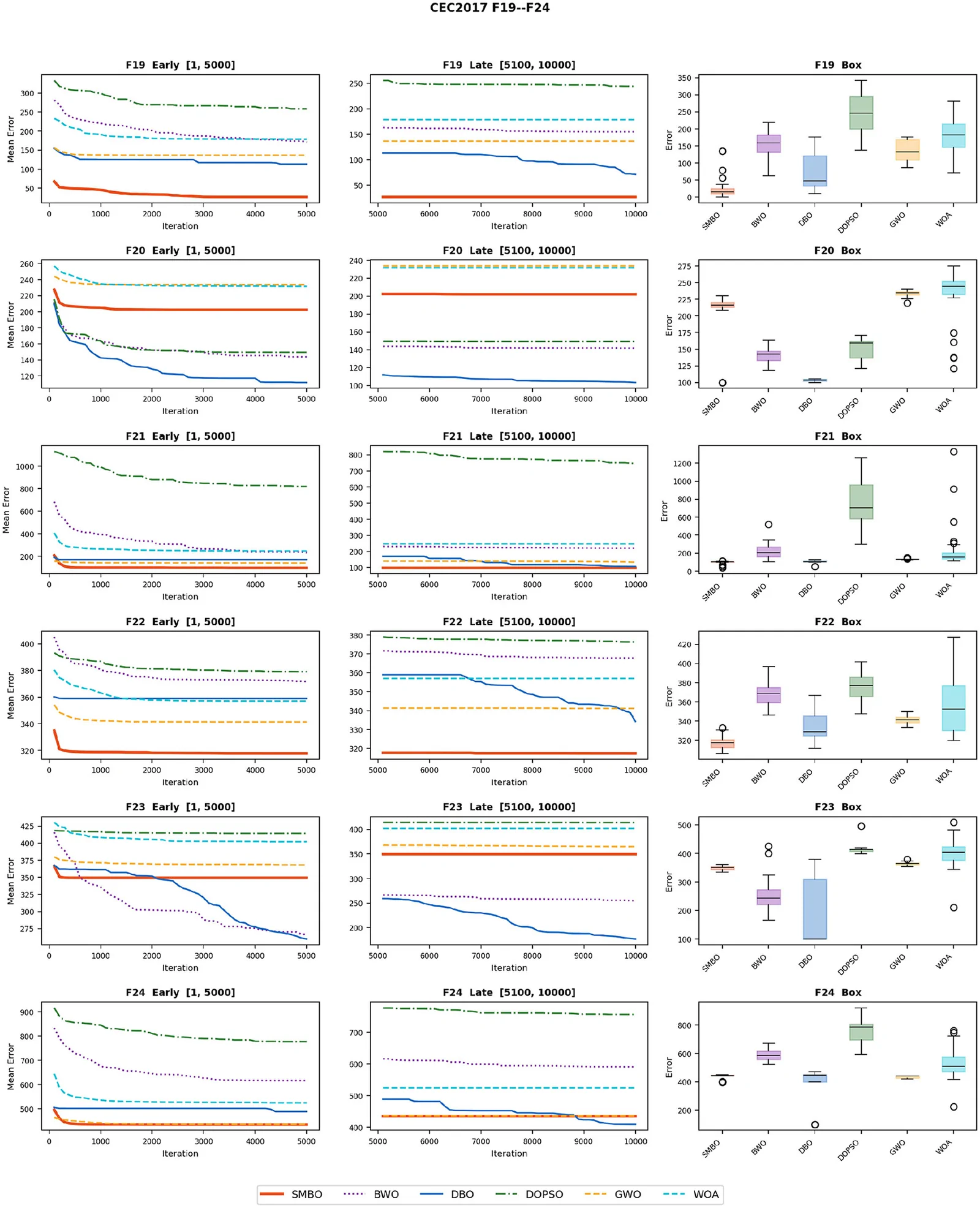
Convergence curves and error distributions of six algorithms on CEC2017 benchmark functions F19–F24.

### 4.3 Ablation study of SMBO

To isolate the contributions of SMBO's three core mechanisms, an ablation study was conducted on the CEC2022 benchmark suite (*D* = 10, Pop = 100, MaxIt = 1, 000, 30 independent runs), comparing the full SMBO with three variants, each replacing exactly one mechanism with a generic, non-adaptive equivalent while leaving all other components unchanged: SMBO-A removes the sinusoidal modulation strategy (SMS), replacing its sinusoidal step-size modulation and Stage-1 sinusoidal perturbation with a linear decay schedule and standard Gaussian noise, respectively; SMBO-B removes Dynamic Behavior Switching (DBS), replacing the probabilistic, overlapping stage-selection scheme with a deterministic hard-threshold schedule; and SMBO-C removes asymmetric information flow (AIF), disabling the Stage-3 archive eject/inject mechanism entirely. Across all 12 functions, the full SMBO ranks best (2.000), followed by SMBO-C (2.333), SMBO-A (2.417), and SMBO-B (3.250). Details are provided in Table 10.

**Table 10:** Ablation study of SMBO on CEC2022 F1–F12 (*D* = 10, Pop = 100, MaxIt = 1, 000, 30 runs).

Func	Metric	SMBO		SMBO-A		SMBO-B		SMBO-C	
		Mean	Std	Mean	Sth	Mean	Sth	Mean	Std
*F*_1_	Results	**1.29E-06**	2.21E-06	5.08E-06	6.35E-06	5.58E+02	5.49E+02	2.08E-05	1.04E-04
	Rank	**1**		2		4		3	
*F*_2_	Results	4.60E+00	3.65E+00	7.16E+00	1.22E+01	6.00E+00	6.71E+00	**4.12E+00**	2.93E+00
	Rank	2		4		3		**1**	
*F*_3_	Results	**5.86E-07**	1.09E-06	2.82E-06	3.68E-06	2.37E-01	2.72E-01	3.37E-02	1.17E-01
	Rank	**1**		2		4		3	
*F*_4_	Results	1.01E+01	4.66E+00	1.11E+01	4.49E+00	**9.09E+00**	3.22E+00	1.03E+01	4.11E+00
	Rank	2		4		**1**		3	
*F*_5_	Results	1.39E+00	2.62E+00	**1.54E-01**	3.50E-01	2.01E+01	1.36E+01	2.32E+00	9.11E+00
	Rank	2		**1**		4		3	
*F*_6_	Results	2.52E+02	7.90E+02	1.39E+02	3.87E+02	**4.15E+01**	1.62E+02	5.17E+01	8.56E+01
	Rank	4		3		**1**		2	
*F*_7_	Results	**2.80E+00**	5.71E+00	6.11E+00	8.63E+00	1.02E+01	9.39E+00	7.42E+00	9.65E+00
	Rank	**1**		2		4		3	
*F*_8_	Results	**9.24E+00**	9.59E+00	1.02E+01	9.85E+00	1.56E+01	6.82E+00	1.27E+01	8.89E+00
	Rank	**1**		2		4		3	
*F*_9_	Results	**2.29E+02**	1.15E-07	2.29E+02	3.49E-07	2.30E+02	1.81E+00	2.29E+02	3.59E-12
	Rank	**1**		2		4		3	
*F*_10_	Results	7.83E+01	4.01E+01	8.14E+01	3.80E+01	7.70E+01	4.86E+01	**7.42E+01**	4.04E+01
	Rank	3		4		2		**1**	
*F*_11_	Results	6.03E+01	7.38E+01	**2.51E+01**	5.61E+01	7.66E+01	6.92E+01	5.02E+01	7.10E+01
	Rank	3		**1**		4		2	
*F*_12_	Results	1.66E+02	1.38E+00	1.66E+02	1.10E+00	1.67E+02	1.99E+00	**1.66E+02**	1.37E+00
	Rank	3		2		4		**1**	
Avg Rank		2.000		2.417		3.250		2.333	

SMBO-A, w/o SMS; SMBO-B, w/o DBS; SMBO-C, w/o AIF. Bold: best Mean or Rank = 1.

To evaluate how SMBO's search capability is affected by population size, we conducted a population-size analysis in which SMBO alone was run on CEC2022 (D = 10, MaxIt = 1,000, 30 independent runs) with population sizes of 10, 30, 50, and 100; the results are summarized in Table 11. SMBO's mean error decreased monotonically with increasing population size across all 12 functions, with no instability or performance degradation observed even at the largest tested population (Pop = 100). This suggests that SMBO remains numerically stable as population size grows.

**Table 11:** SMBO with population 10, 30, 50, 100 test on CEC2022 F1–F12 (*D* = 10, MaxIt = 1, 000, 30 runs), Pop ∈{10, 30, 50, 100}.

Func	Metric	Pop = 10	Pop = 30	Pop = 50	Pop = 100
*F*_1_	Mean	4.12E+03	1.78E+02	1.81E+00	1.29E-06
	Std	4.99E+03	4.30E+02	8.37E+00	2.21E-06
*F*_2_	Mean	1.88E+01	1.11E+01	4.76E+00	4.60E+00
	Std	2.82E+01	1.98E+01	3.33E+00	3.65E+00
*F*_3_	Mean	1.42E+01	8.22E-02	9.49E-05	5.86E-07
	Std	8.46E+00	2.32E-01	2.28E-04	1.09E-06
*F*_4_	Mean	3.23E+01	1.95E+01	1.43E+01	1.01E+01
	Std	1.00E+01	7.96E+00	5.89E+00	4.66E+00
*F*_5_	Mean	4.32E+02	5.58E+01	1.35E+01	1.39E+00
	Std	2.70E+02	7.52E+01	2.01E+01	2.62E+00
*F*_6_	Mean	2.13E+03	1.69E+03	1.47E+03	2.52E+02
	Std	1.85E+03	1.71E+03	1.63E+03	7.90E+02
*F*_7_	Mean	6.46E+01	2.27E+01	1.28E+01	2.80E+00
	Std	4.50E+01	1.78E+01	9.49E+00	5.71E+00
*F*_8_	Mean	4.68E+01	1.97E+01	1.60E+01	9.24E+00
	Std	5.72E+01	3.54E+00	7.57E+00	9.59E+00
*F*_9_	Mean	2.47E+02	2.29E+02	2.29E+02	2.29E+02
	Std	4.36E+01	2.95E-05	5.67E-06	1.15E-07
*F*_10_	Mean	1.95E+02	1.10E+02	8.16E+01	7.83E+01
	Std	1.14E+02	5.54E+01	3.79E+01	4.01E+01
*F*_11_	Mean	1.89E+02	1.16E+02	6.53E+01	6.03E+01
	Std	1.12E+02	8.40E+01	7.47E+01	7.38E+01
*F*_12_	Mean	1.84E+02	1.69E+02	1.67E+02	1.66E+02
	Std	1.50E+01	3.87E+00	2.80E+00	1.38E+00

### 4.4 Experimental results with brain cancer images

In the brain cancer image clustering test, 1,505 brain magnetic resonance images are used. The dataset contains four classes: Glioma (373 images), Meningioma (363 images), Normal (396 images), and Pituitary (373 images). Details are shown in Figure 8. To evaluate the robustness of SMBO with respect to the choice of feature representation, three widely used pre-trained convolutional neural network backbones are used for feature extraction: ResNet50, VGG16, and DenseNet121, each pre-trained on ImageNet and used as a fixed feature extractor without fine-tuning. For each backbone, the penultimate-layer activations (2,048-dimensional for ResNet50, 4,096-dimensional for VGG16, and 1,024-dimensional for DenseNet121) are standardized and reduced via Principal Component Analysis (PCA) to the top-10 principal components, which serve as the clustering feature space for all subsequent experiments. In this study, the objective function is formulated as the complement of clustering Purity, defined as *f* = 1 − Purity, which converts the inherently maximization-oriented clustering metric into a minimization problem compatible with the standard metaheuristic optimization interface. Specifically, each candidate solution encodes the coordinates of *K* = 4 cluster centers in the PCA-reduced feature space, and cluster membership is determined by minimum Euclidean distance assignment. The Purity of a given partition is computed as $\text{Purity}=\frac{1}{N}\overset{K}{\underset{k=1}{\sum }}\underset{j}{\max}|C_{k}\cap T_{j}|$, where *N* denotes the total number of samples, *C*_*k*_ represents the set of samples assigned to the *k*-th cluster, and *T*_*j*_ denotes the set of samples belonging to the *j*-th ground-truth class.

**Figure 5:**
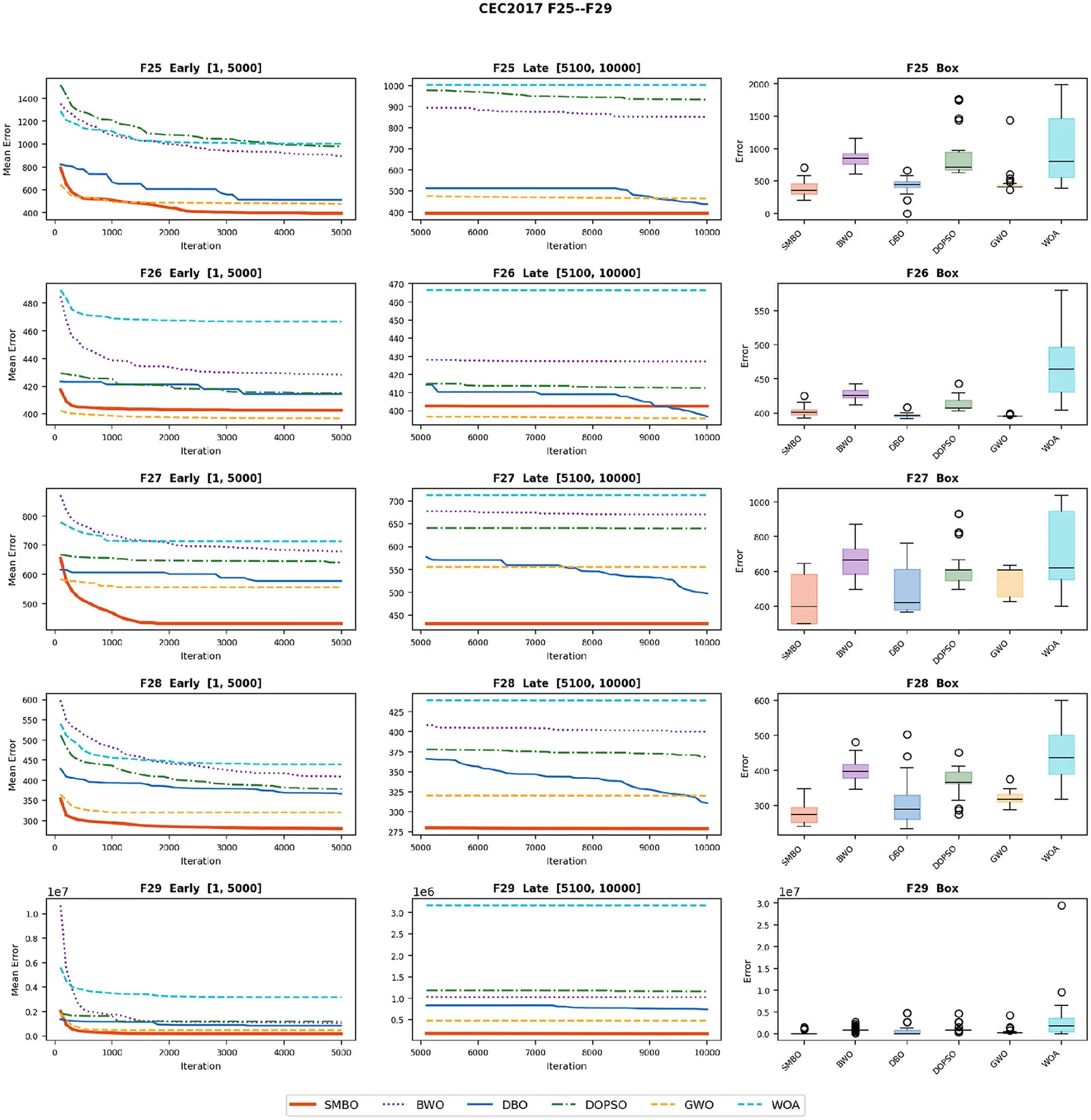
Convergence curves and distributions of six algorithms on CEC2017 benchmark functions F25–F29.

In the brain cancer image clustering test, 1,505 brain magnetic resonance images are used. The dataset contains four classes: Glioma (373 images), Meningioma (363 images), Normal (396 images), and Pituitary (373 images). Details are shown in Figure 8. To evaluate the robustness of SMBO with respect to the choice of feature representation, three widely used pre-trained convolutional neural network backbones are employed for feature extraction: ResNet50, VGG16, and DenseNet121, each pre-trained on ImageNet and used as a fixed feature extractor without fine-tuning. For each backbone, the penultimate-layer activations (2,048-dimensional for ResNet50, 4,096-dimensional for VGG16, and 1,024-dimensional for DenseNet121) are standardized and reduced via Principal Component Analysis (PCA) to the top-10 principal components, which serve as the clustering feature space for all subsequent experiments. In this study, the objective function is formulated as the complement of clustering Purity, defined as *f* = 1 − Purity, which converts the inherently maximization-oriented clustering metric into a minimization problem compatible with the standard metaheuristic optimization interface. Specifically, each candidate solution encodes the coordinates of *K* = 4 cluster centers in the PCA-reduced feature space, and cluster membership is determined by minimum Euclidean distance assignment. The Purity of a given partition is computed as $\text{Purity}=\frac{1}{N}\overset{K}{\underset{k=1}{\sum }}\underset{j}{\max}|C_{k}\cap T_{j}|$, where *N* denotes the total number of samples, *C*_*k*_ represents the set of samples assigned to the *k*-th cluster, and *T*_*j*_ denotes the set of samples belonging to the *j*-th ground-truth class.

Table 12 reports the clustering performance of SMBO on the brain cancer MRI dataset across three feature-extraction backbones (ResNet50, VGG16, DenseNet121 + PCA, Top-10, *K* = 4, Pop = 30, MaxIt = 1, 000, 10 independent runs). On ResNet50, SMBO attains a Purity of 0.8006, an NMI of 0.5293, and an ARI of 0.5561. On VGG16, SMBO reaches a Purity of 0.7821, an NMI of 0.4999, and an ARI of 0.5274. On DenseNet121, SMBO achieves its best overall result, with a Purity of 0.8636, an NMI of 0.6733, and an ARI of 0.6881. Figure 9 shows the corresponding convergence behavior: across all three backbones, SMBO's mean fitness (1 − Purity) curve descends steadily and continues to improve throughout the full 1,000-iteration budget, and the boxplots of final Purity over the 10 independent runs show a tightly distributed spread (e.g., an interquartile range under 0.02 Purity on DenseNet121), reflecting consistent run-to-run stability. Table 13 reports the clustering performance results of SMBO against K-means++ and Gaussian Mixture Model (GMM).

**Table 12:** Clustering performance (Purity/NMI/ARI) across three feature-extraction backbones (ResNet50, VGG16, DenseNet121 + PCA, Top-10, *K* = 4, Pop = 30, MaxIt = 1, 000, 10 runs).

Algorithm	ResNet50			VGG16			DenseNet121		
	Purity	NMI	ARI	Purity	NMI	ARI	Purity	NMI	ARI
SMBO	**0.8006**	**0.5293**	**0.5561**	**0.7821**	**0.4999**	**0.5274**	**0.8636**	**0.6733**	**0.6881**
DBO	0.7060	0.4056	0.4200	0.6981	0.4275	0.4350	0.7783	0.5230	0.5354
GWO	0.6939	0.4380	0.4341	0.6899	0.4038	0.4193	0.8064	0.5685	0.5809
BWO	0.6705	0.3602	0.3580	0.6765	0.3594	0.3717	0.7107	0.4143	0.4187
WOA	0.6133	0.3037	0.3006	0.5773	0.2547	0.2508	0.6590	0.3676	0.3635
DOPSO	0.6890	0.3620	0.3701	0.6555	0.3611	0.3676	0.7658	0.4990	0.5119

Bold: best value per backbone column.

**Figure 6:**
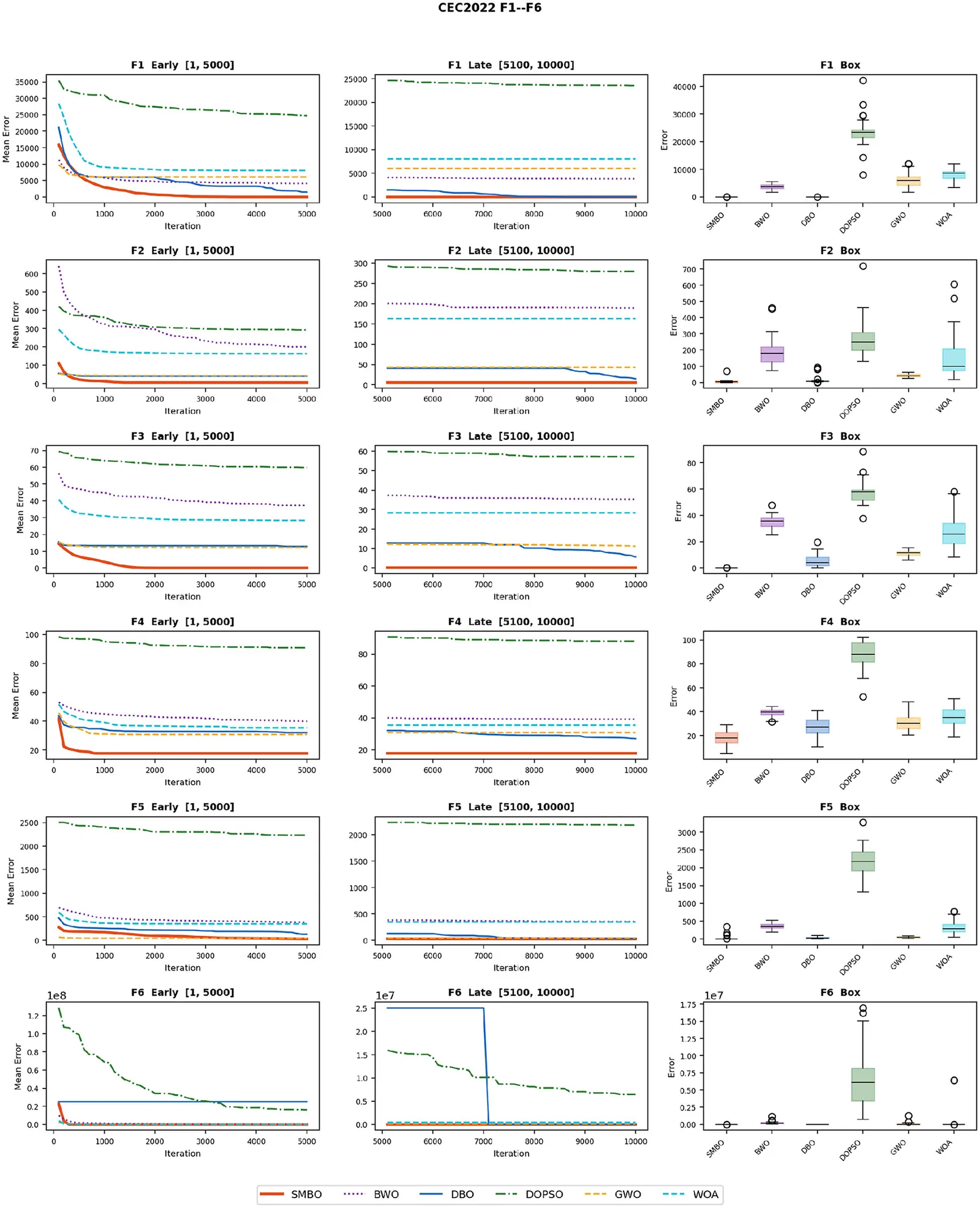
Convergence curves and error distributions of six algorithms on CEC2022 benchmark functions F1–F6.

**Figure 7:**
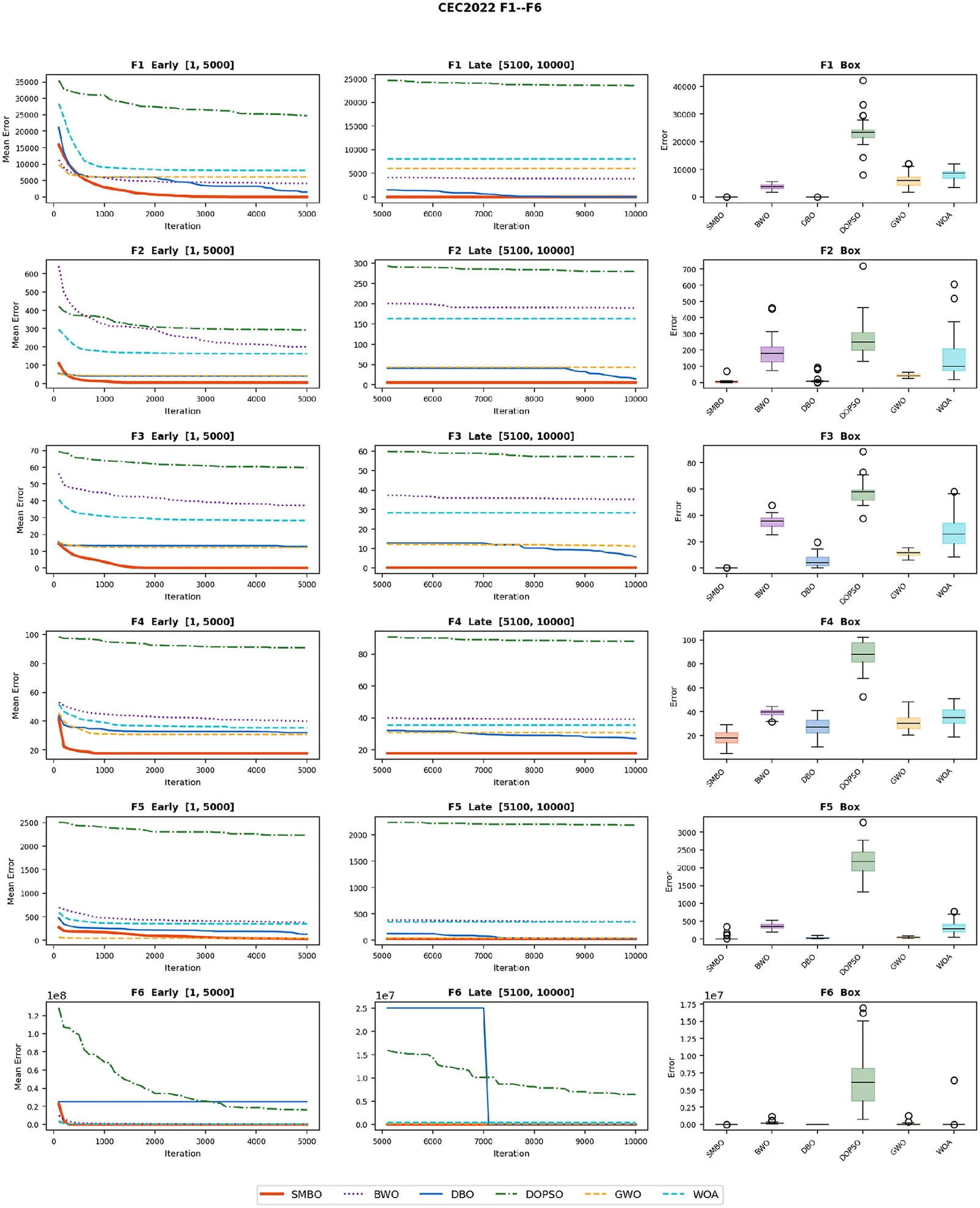
Convergence curves and error distributions of six algorithms on CEC2022 benchmark functions F7–F12.

**Figure 8:**
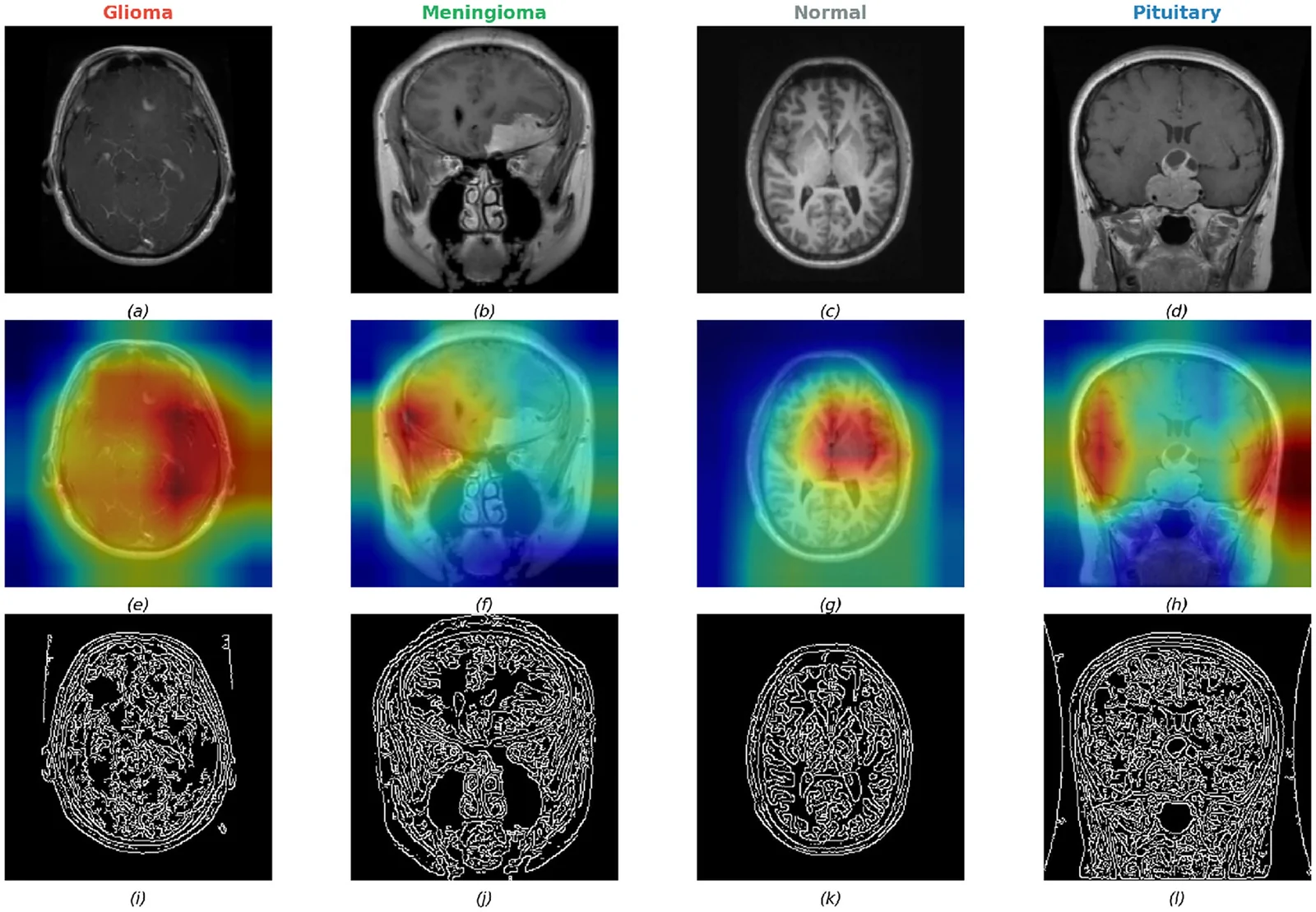
Feature details for brain cancer MRI images: **(a–d)** original MRI images of four categories (Glioma, Meningioma, Normal, Pituitary); **(e–h)** Grad-CAM activation maps generated from the ResNet50 last convolutional layer, highlighting discriminative regions; **(i–l)** Canny edge detection results revealing structural boundaries of tumor morphology.

**Figure 9:**
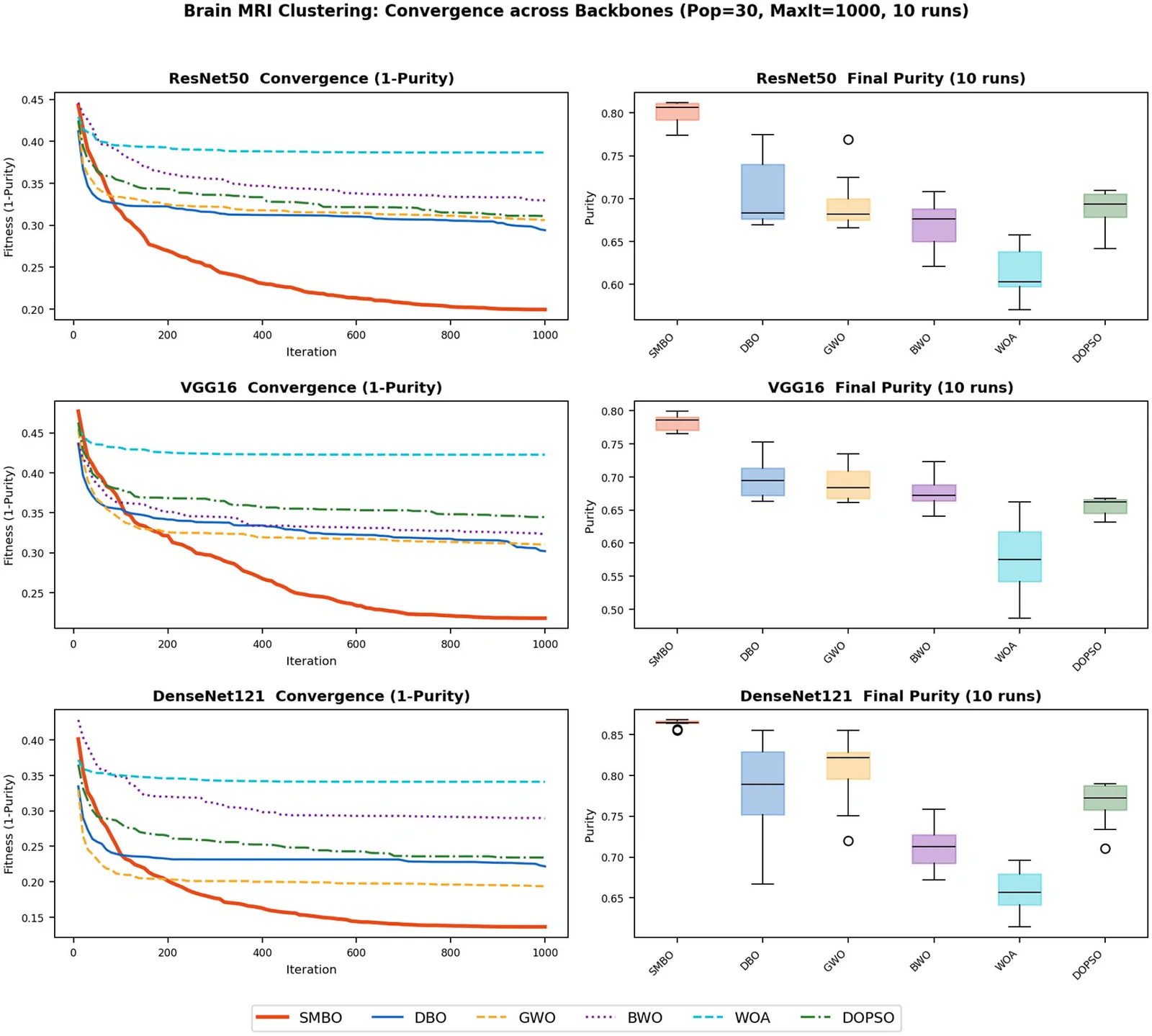
Convergence behavior of SMBO and five baseline algorithms (DBO, GWO, BWO, WOA, DOPSO) on the brain cancer MRI clustering task across three feature-extraction backbones (ResNet50, VGG16, DenseNet121 + PCA, Top-10, *K* = 4, Pop = 30, MaxIt = 1, 000, 10 runs).

**Table 13:** Clustering performance (Purity/NMI/ARI) of SMBO vs. K-means++ and GMM, on the same PCA-reduced features used in Table 12 (ResNet50, VGG16, DenseNet121 + PCA, Top-10, *K* = 4, 10 runs).

Backbone	Method	Purity	NMI	ARI
ResNet50	SMBO	**0.8006**	**0.5293**	**0.5561**
	K-means++	0.4672	0.1868	0.1668
	GMM	0.4819	0.2238	0.2014
VGG16	SMBO	**0.7821**	**0.4999**	**0.5274**
	K-means++	0.4823	0.1848	0.1715
	GMM	0.4920	0.1945	0.1741
DenseNet121	SMBO	**0.8636**	**0.6733**	**0.6881**
	K-means++	0.4720	0.1978	0.1760
	GMM	0.5527	0.3169	0.2574

K-means++/GMM use *n*_*init* = 10 internal restarts per run. Bold: best value per backbone.

## 5 Discussion

Across the numerical benchmarks, SMBO achieved the lowest average rank among the six compared algorithms in every setting: CEC2017 at *D* = 10 (1.38) and *D* = 100 (1.10), and CEC2022 at *D* = 10 (1.33). Friedman tests confirmed statistically significant differences in average rank in all three cases (*p* < 0.001). Pairwise Wilcoxon rank-sum tests show this advantage is broad against BWO, DOPSO, GWO, and WOA, while DBO remains a comparatively strong baseline at *D* = 10; DBO's competitiveness diminishes at *D* = 100, where SMBO's margin over it widens. The ablation study isolating SMBO's three core mechanisms found that removing dynamic behavior switching (DBS) substantially reduced solution quality on average, while removing the sinusoidal modulation strategy (SMS) or the asymmetric information flow (AIF) produced smaller, more function-dependent effects. On the brain cancer MRI clustering task, SMBO provided high-precision solutions across all three feature extraction backbones (ResNet50, VGG16, DenseNet121).

These results are consistent with the intended roles of SMBO's core mechanisms. DBS governs the overall exploration-exploitation schedule by switching search behavior based on iteration progress and fitness feedback rather than a fixed threshold. SMS provides self-adaptive step-size control that avoids the uniform shrinkage responsible for premature convergence, while AIF sustains diversity by restricting information transfer to flow one-way from the elite pool to the exploration pool, preventing the exploration pool from collapsing toward elite solutions. Together, these mechanisms account for the more targeted, function-dependent gains observed in the ablation study and for SMBO's stable, low-variance behavior on the brain MRI clustering task.

Several limitations should be noted. First, the numerical benchmark evaluations do not cover larger-scale or real-world engineering optimization problems, so SMBO's scaling behavior and practical applicability remain to be verified. Second, the clustering evaluation is currently limited to brain tumor MRI data and has not been extended to other disease conditions or imaging modalities, limiting the generality of the conclusions.

## 6 Conclusions

This study presented the Sinusoidal Modulation Bi-population Optimizer (SMBO), a metaheuristic algorithm that integrates sinusoidal modulation step-size control, dynamic behavior switching, and asymmetric information flow to balance exploration and exploitation without manual threshold tuning. On the CEC2017 (*D* = 10, *D* = 100) and CEC2022 (*D* = 10) benchmark suites, SMBO achieved the lowest average rank among six compared algorithms in every setting, and Friedman and Wilcoxon rank-sum tests confirmed that these differences were statistically significant. An ablation study further showed that dynamic behavior switching substantially affects SMBO's performance, whereas the sinusoidal modulation strategy and asymmetric information flow provide smaller, function-dependent refinements. A population size analysis confirmed that SMBO remains numerically stable as population size increases, with no divergence or degradation observed.

Future research will extend the evaluation to larger-scale and real-world engineering optimization problems, apply the clustering pipeline to additional disease conditions and imaging modalities, and incorporate a fully unsupervised clustering criterion in place of the current label-guided fitness function.

## Data Availability

The raw data supporting the conclusions of this article will be made available by the authors, without undue reservation.

## References

[B1] Abd El-Hafeez T. Tarek M. Sayed A. (2025). Optimizing YOLOv11 for automated classification of breast cancer in medical images. Sci. Rep. 15:37170. doi: 10.1038/s41598-025-24850-7 PMC12550060 41131122

[B2] Akter A. Nosheen N. Ahmed S. Hossain M. Yousuf M. A. Almoyad M. A. A. . (2024). Robust clinical applicable CNN and U-Net based algorithm for MRI classification and segmentation for brain tumor. Expert Syst. Appl. 238:122347. doi: 10.1016/j.eswa.2023.122347

[B3] Allahem H. El-Ghany S. A. Abd El-Aziz A. A. Aldughayfiq B. Alshammeri M. Alamri M. (2025). A hybrid model of feature extraction and dimensionality reduction using ViT, PCA, and random forest for multi-classification of brain cancer. Diagnostics 15:1392. doi: 10.3390/diagnostics15111392 PMC12153998 40506964

[B4] Andrijanova A. Bugovecka L. Isajevs S. Erts D. Malinovskis U. Liepins A. (2025). Machine learning for lung cancer subtype classification: combining clinical, histopathological, and biophysical features. Diagnostics 15:127. doi: 10.3390/diagnostics15020127 PMC11764335 39857011

[B5] Askari Q. Younas I. Saeed M. (2020). Political Optimizer: a novel socio-inspired meta-heuristic for global optimization. Knowl.-Based Syst. 195:105709. doi: 10.1016/j.knosys.2020.105709

[B6] Celik M. Inik O. (2024). Development of hybrid models based on deep learning and optimized machine learning algorithms for brain tumor Multi-Classification. Expert Syst. Appl. 238:122159. doi: 10.1016/j.eswa.2023.122159

[B7] Deng L. Liu S. (2023). Snow ablation optimizer: a novel metaheuristic technique for numerical optimization and engineering design. Expert Syst. Appl. 225:120069. doi: 10.1016/j.eswa.2023.120069

[B8] Dutta T. K. Nayak D. R. Zhang Y. D. (2024). ARM-Net: attention-guided residual multiscale CNN for multiclass brain tumor classification using MR images. Biomed. Signal Process. Control 87:105421. doi: 10.1016/j.bspc.2023.105421

[B9] Goceri E. (2024). Vision transformer based classification of gliomas from histopathological images. Expert Syst. Appl. 241:122672. doi: 10.1016/j.eswa.2023.122672

[B10] Guo J. Li Y. Yuan Q. Sun G. Di Y. Sato Y. (2026). A dual oscillatory particle swarm optimization for tomato disease classification. Peer J. Comput. Sci. 12:e3566. doi: 10.7717/peerj-cs.3566

[B11] Guo J. Sato Y. (2018) “A dynamic reconstruction bare bones particle swarm optimization algorithm,” in 2018 IEEE Congress on Evolutionary Computation (CEC) (Rio de Janeiro), 1–6. doi: 10.1109/CEC.2018.8477883

[B12] Guo J. Sato Y. (2019). A fission-fusion hybrid bare bones particle swarm optimization algorithm for single-objective optimization problems. Appl. Intell. 49, 3641–3651. doi: 10.1007/s10489-019-01474-9

[B13] Guo J. Shi B. Yan K. Di Y. Tang J. Xiao H. . (2022). A twinning bare bones particle swarm optimization algorithm. PLoS ONE 17:e0267197. doi: 10.1371/journal.pone.0267197 PMC9060357 35500006

[B14] Guo J. Ye W. Wang D. He Z. Yan Z. Sato M. . (2024a). A Novel Snow Leopard Optimization for High-Dimensional Feature Selection Problems. Sensors 24:7161. doi: 10.3390/s24227161 PMC11598119 39598941

[B15] Guo J. Yuan H. Shi B. Zheng X. Zhang Z. Li H. . (2024b). A novel breast cancer image classification model based on multiscale texture feature analysis and dynamic learning. Sci. Rep. 14:7216. doi: 10.1038/s41598-024-57891-5 PMC10973442 38538814

[B16] Guo J. Zhou G. Di Y. Shi B. Yan K. Sato Y. (2023a). A bare-bones particle swarm optimization with crossed memory for global optimization. IEEE Access 11, 31549–31568. doi: 10.1109/ACCESS.2023.3250228

[B17] Guo J. Zhou G. Yan K. Di Y. Sato Y. He Z. . (2025). A deep backtracking bare bones particle swarm optimisation algorithm for high dimensional nonlinear functions. CAAI Trans. Intell. Technol. 10, 1501–152. doi: 10.1049/cit2.70028

[B18] Guo J. Zhou G. Yan K. Sato Y. Di Y. (2023b). Pair barracuda swarm optimization algorithm: a natural-inspired metaheuristic method for high dimensional optimization problems. Sci. Rep. 13:18314. doi: 10.1038/s41598-023-43748-w PMC10600174 37880214

[B19] Guo J. Zhou G. Yan K. Shi B. Di Y. Sato Y. (2023c). A novel hermit crab optimization algorithm. Sci. Rep. 13, 1–26. doi: 10.1038/s41598-023-37129-6 PMC10279639 37337020

[B20] Hamza M. N. Koziel S. Pietrenko-Dabrowska A. (2024). Design and experimental validation of a metamaterial-based sensor for microwave imaging in breast, lung, and brain cancer detection. Sci. Rep. 14:16177. doi: 10.1038/s41598-024-67103-9 PMC11246499 39003304

[B21] Hayum A. A. Daniel Shadrach F. (2025). Optimizing breast cancer classification based on cat swarm-enhanced ensemble neural network approach for improved diagnosis and treatment decisions. Sci. Rep. 15:33740. doi: 10.1038/s41598-025-95481-1 PMC12485177 41028518

[B22] Hossain S. Chakrabarty A. Gadekallu T. R. Alazab M. Piran M. J. (2024). Vision transformers, ensemble model, and transfer learning leveraging explainable ai for brain tumor detection and classification. IEEE J. Biomed. Health Informat. 28, 1261–1272. doi: 10.1109/JBHI.2023.3266614 37043319

[B23] Islam M. M. Talukder M. A. Uddin M. A. Akhter A. Khalid M. (2024a). BrainNet: precision brain tumor classification with optimized EfficientNet architecture. Int. J. Intell. Syst. 2024:3583612. doi: 10.1155/2024/3583612

[B24] Islam M. R. Rahman M. M. Ali M. S. Nafi A. A. N. Alam M. S. Godder T. K. . (2024b). Enhancing breast cancer segmentation and classification: an ensemble deep convolutional neural network and U-net approach on ultrasound images. Mach. Learn. Applic. 16:100555. doi: 10.1016/j.mlwa.2024.100555

[B25] Kaddes M. Ayid Y. M. Elshewey A. M. Fouad Y. (2025). Breast cancer classification based on hybrid CNN with LSTM model. Sci. Rep. 15:4409. doi: 10.1038/s41598-025-88459-6 PMC11799331 39910136

[B26] Kang J. Ullah Z. Gwak J. (2021). Mri-based brain tumor classification using ensemble of deep features and machine learning classifiers. Sensors 21:2222. doi: 10.3390/s21062222 PMC8004778 33810176

[B27] Lang Y. Gao Y. (2025). Dream Optimization Algorithm (DOA): a novel metaheuristic optimization algorithm inspired by human dreams and its applications to real-world engineering problems. Comput. Methods Appl. Mech. Eng. 436:117718. doi: 10.1016/j.cma.2024.117718

[B28] Ling Y. Wang Y. Dai W. Yu J. Liang P. Kong D. (2024). MTANet: multi-task attention network for automatic medical image segmentation and classification. IEEE Trans. Med. Imaging 43, 674–685. doi: 10.1109/TMI.2023.3317088 37725719

[B29] Ma H. Shen S. Yu M. Yang Z. Fei M. Zhou H. (2019). Multi-population techniques in nature inspired optimization algorithms: a comprehensive survey. Swarm Evolut. Comput. 44, 365–387. doi: 10.1016/j.swevo.2018.04.011

[B30] Ma Z. Wu G. Suganthan P. N. Song A. Luo Q. (2023). Performance assessment and exhaustive listing of 500+ nature-inspired metaheuristic algorithms. Swarm Evolut. Comput. 77:101248. doi: 10.1016/j.swevo.2023.101248

[B31] Mafarja M. Mirjalili S. (2018). Whale optimization approaches for wrapper feature selection. Appl. Soft Comput. 62, 441–453. doi: 10.1016/j.asoc.2017.11.006

[B32] Mansouri H. Elkhanchouli K. Elghouate N. Bencherqui A. Tahiri M. A. Karmouni H. . (2025). A modified black-winged kite optimizer based on chaotic maps for global optimization of real-world applications. Knowl.-Based Syst. 318:113558. doi: 10.1016/j.knosys.2025.113558

[B33] Mercaldo F. Brunese L. Martinelli F. Santone A. Cesarelli M. (2023a). Explainable convolutional neural networks for brain cancer detection and localisation. Sensors 23:7614. doi: 10.3390/s23177614 PMC10490676 37688069

[B34] Mercaldo F. Brunese L. Martinelli F. Santone A. Cesarelli M. (2023b). Object detection for brain cancer detection and localization. Appl. Sci. 13:9158. doi: 10.3390/app13169158 PMC10490676 37688069

[B35] Mirjalili S. Lewis A. (2016). The whale optimization algorithm. Adv. Eng. Softw. 95, 51–67. doi: 10.1016/j.advengsoft.2016.01.008

[B36] Mirjalili S. Mirjalili S. M. Lewis A. (2014). Grey wolf optimizer. Adv. Eng. Soft. 69, 46–61. doi: 10.1016/j.advengsoft.2013.12.007

[B37] Murtaza G. Shuib L. Abdul Wahab A. W. Mujtaba G. Mujtaba G. Nweke H. F. . (2020). Deep learning-based breast cancer classification through medical imaging modalities: state of the art and research challenges. Artif. Intell. Rev. 53, 1655–1720. doi: 10.1007/s10462-019-09716-5

[B38] Nagarani N. Karthick R. Sandra Carmel Sophia M. Binda M. B. (2024). Self-attention based progressive generative adversarial network optimized with momentum search optimization algorithm for classification of brain tumor on MRI image. Biomed. Signal Process. Control 88:105597. doi: 10.1016/j.bspc.2023.105597

[B39] Peraza-Vázquez H. Pe na-Delgado A. Merino-Trevi no M. Morales-Cepeda A. B. Sinha N. (2024). A novel metaheuristic inspired by horned lizard defense tactics. Artif. Intell. Rev. 57, 1–65. doi: 10.1007/s10462-023-10653-7

[B40] Piotrowski A. P. (2018). L-shade optimization algorithms with population-wide inertia. Inf. Sci. 468, 117–141. doi: 10.1016/j.ins.2018.08.030

[B41] Reddy C. K. K. Reddy P. A. Janapati H. Assiri B. Shuaib M. Alam S. . (2024). A fine-tuned vision transformer based enhanced multi-class brain tumor classification using MRI scan imagery. Front. Oncol. 14:1400341. doi: 10.3389/fonc.2024.1400341 PMC11291226 39091923

[B42] Shoaib M. R. Zhao J. Emara H. M. Mubarak A. S. Omer O. A. Abd El-Samie F. E. . (2025). Improving brain tumor classification: an approach integrating pre-trained CNN models and machine learning algorithms. Heliyon 11:1714. doi: 10.1016/j.heliyon.2024.e33471

[B43] Srinivasan S. Francis D. Mathivanan S. K. Rajadurai H. Shivahare B. D. Shah M. A. (2024). A hybrid deep CNN model for brain tumor image multi-classification. BMC Med. Imaging 24:21. doi: 10.1186/s12880-024-01195-7 PMC10799524 38243215

[B44] Tanabe R. Fukunaga A. (2013). “Success-history based parameter adaptation for differential evolution,” in 2013 IEEE congress on evolutionary computation (Cancun: IEEE), 71–78. doi: 10.1109/CEC.2013.6557555

[B45] Tanabe R. Fukunaga A. S. (2014). “Improving the search performance of SHADE using linear population size reduction,” in 2014 IEEE congress on evolutionary computation (CEC) (Beijing: IEEE), 1658–1665. doi: 10.1109/CEC.2014.6900380

[B46] Ting F. F. Tan Y. J. Sim K. S. (2019). Convolutional neural network improvement for breast cancer classification. Expert Syst. Appl. 120, 103–115. doi: 10.1016/j.eswa.2018.11.008

[B47] Wan Y. Wang M. Ye Z. Lai X. (2016). A feature selection method based on modified binary coded ant colony optimization algorithm. Appl. Soft Comput. 49, 248–258. doi: 10.1016/j.asoc.2016.08.011

[B48] Wang J. Lu S. Y. Wang S. H. Zhang Y. D. (2024). RanMerFormer: randomized vision transformer with token merging for brain tumor classification. Neurocomputing 573:127216. doi: 10.1016/j.neucom.2023.127216

[B49] Wang M. Wan Y. Ye Z. Lai X. (2017). Remote sensing image classification based on the optimal support vector machine and modified binary coded ant colony optimization algorithm. Inf. Sci. 402, 50–68. doi: 10.1016/j.ins.2017.03.027

[B50] Wu Y. Chen B. Zeng A. Pan D. Wang R. Zhao S. (2022). Skin cancer classification with deep learning: a systematic review. Front. Oncol. 12:893972. doi: 10.3389/fonc.2022.893972 PMC9327733 35912265

[B51] Xiao H. Guo J. Shi B. Di Y. Pan C. Yan K. . (2022). A twinning memory bare-bones particle swarm optimization algorithm for no-linear functions. IEEE Access 11, 25768–25785. doi: 10.1109/ACCESS.2022.3222530

[B52] Xue J. Shen B. (2023). Dung beetle optimizer: a new meta-heuristic algorithm for global optimization. J. Supercomput. 79, 7305–7336. doi: 10.1007/s11227-022-04959-6

[B53] Yagappan A. J. Karuppiah H. Muthusamy M. Kannaiah S. K. (2025). Optimizing lung cancer classification using transformer and Gooseneck Barnacle Optimization. Expert Syst. Appl. 282:127413. doi: 10.1016/j.eswa.2025.127413

[B54] Yang G. Luo S. Greer P. (2025). Boosting skin cancer classification: a multi-scale attention and ensemble approach with vision transformers. Sensors 25:2479. doi: 10.3390/s25082479 PMC12030980 40285168

[B55] Ye A. Z. Li B. R. Zhou C. W. Wang D. M. Mei E. M. Shu F. Z. . (2023). High-dimensional feature selection based on improved binary ant colony optimization combined with hybrid rice optimization algorithm. Int. J. Intell. Syst. 2023:1444938. doi: 10.1155/2023/1444938

[B56] Zhong C. Li G. Meng Z. (2022). Beluga whale optimization: a novel nature-inspired metaheuristic algorithm. Knowl.-Based Syst. 251:109215. doi: 10.1016/j.knosys.2022.109215

